# PanDelos: a dictionary-based method for pan-genome content discovery

**DOI:** 10.1186/s12859-018-2417-6

**Published:** 2018-11-30

**Authors:** Vincenzo Bonnici, Rosalba Giugno, Vincenzo Manca

**Affiliations:** Department of Computer Science, University of Verona, Strada le Grazie, 15, Verona, 37134 Italy

**Keywords:** Pan-genome, Distant genomes, k-mer dictionary

## Abstract

**Background:**

Pan-genome approaches afford the discovery of homology relations in a set of genomes, by determining how some gene families are distributed among a given set of genomes. The retrieval of a complete gene distribution among a class of genomes is an NP-hard problem because computational costs increase with the number of analyzed genomes, in fact, all-against-all gene comparisons are required to completely solve the problem. In presence of phylogenetically distant genomes, due to the variability introduced in gene duplication and transmission, the task of recognizing homologous genes becomes even more difficult. A challenge on this field is that of designing fast and adaptive similarity measures in order to find a suitable pan-genome structure of homology relations.

**Results:**

We present PanDelos, a stand alone tool for the discovery of pan-genome contents among phylogenetic distant genomes. The methodology is based on information theory and network analysis. It is parameter-free because thresholds are automatically deduced from the context. PanDelos avoids sequence alignment by introducing a measure based on *k*-mer multiplicity. The *k*-mer length is defined according to general arguments rather than empirical considerations. Homology candidate relations are integrated into a global network and groups of homologous genes are extracted by applying a community detection algorithm.

**Conclusions:**

PanDelos outperforms existing approaches, Roary and EDGAR, in terms of running times and quality content discovery. Tests were run on collections of real genomes, previously used in analogous studies, and in synthetic benchmarks that represent fully trusted golden truth. The software is available at https://github.com/GiugnoLab/PanDelos.

**Electronic supplementary material:**

The online version of this article (10.1186/s12859-018-2417-6) contains supplementary material, which is available to authorized users.

## Background

A pan-genome can be abstractly considered as a structure defined on a set of genomes. The structure is built by identifying groups of homologous genes [1]. Two genes are homologous if they share a common ancestral gene. Homologous genes can be distinguished into paralogous, when homology occurs within the same genome, or orthologous, when homology occurs between different genomes. We call pan-genome content discovery the determination of homologous groups within a collection of genomes.

Different mechanisms are involved in gene transmission. Paralogy is linked to sequence duplication within the same genome. Orthology is associated to a “vertical” transmission. It happens among genomes in the same lineage and involves most of the genetic contents. On the contrary, “horizontal” transmission occurs between genomes of organisms of different lineages, involving one or few genes. Genes present in every genome are *core* genes of the pan-genome and they may be involved in essential living functionalities. Sequences shared by a subset of genomes are referred as *dispensable* and they represent variable features. *Singleton* genes are present only in one genome and represent some genome-specific functionality. The collective analyses of all the genes is developed for many specific interests, for example, for the study of a bacterial strain of a given species [2, 3]. Pan-genome analyses found many application in clinical studies [4, 5], for example they help in identifying drug-target genes in clinical studies [6, 7], or in exploring phylogenetic lineages of bacteria [8] that can be linked to strain-specific disease phenotypes [9].

Approaches to pan-genome content discovery need to take into account that gene duplication and transmission may introduce sequence alterations [10–13]. The variations make the task of recognizing homologous genes difficult, especially when ancestor genomes are no more available. Core genes are often under strong evolutionary selection, thus their sequences are transmitted almost without any alteration. The amount of variations affecting dispensable genes varies and the similarity between homologous sequences tends to decrease according to their phylogenetic distance. When closely related organisms are analyzed, reasonable thresholds on sequences similarity are applied to recognize gene families. However, when distant genomes are compared, global thresholds result less feasible. Suitable notions are needed to define adaptive thresholds especially when they present non-uniform phylogenetic distances.

The discovery of a pan-genome content is an NP-hard problem [14], and the complexity of the analysis is proportional to the number of input genomes. This is mainly due to the fact that all-against-all comparisons between gene sets are required to solve the task. State of art tools for pan-genome analysis are Roary [15] and EDGAR [16]. They use some heuristics to reduce the computational requirements in the definitions of thresholds for sequence alignments and the number of comparisons necessary in their procedures. Both approaches are based on a largely-used strategy that searches for *reciprocally* most similar genes between compared genomes [17, 18].

Roary combines an approach for clustering gene sequences (CD-HIT) with a procedure based on reciprocal BLAST alignments. CD-HIT [19] clustering counts the presence of *k*-mers, substrings of length *k*, among the analyzed sequences at different values of *k*. The results of CD-HIT are merged with normalized BLAST scores [20] and clustered via the MCL algorithm [21]. The Roary’s procedure requires intensively tuning of user-defined parameters to set the thresholds for discarding low homology values. Parameters are set globally, making Roary best performing on closely related genomes.

EDGAR uses adaptive thresholds depending on the distribution of BLAST gene scores. The retrieval of a distribution is made feasible by the normalization of alignment scores. The normalization is performed by fixing the self-alignment score of a sequence as the maximum one. The approach results suitable for comparing genomes with a considerable phylogenetic distance, but some disadvantages arise. It requires an expensive amount of sequence alignments, in fact for each 1-vs-1 genome comparisons, the complete gene sets of the two genomes must be cross-aligned. EDGAR chooses the threshold on the minimum feasible score by computing the distribution of the normalized gene blast of all scores. Scores are summed up and represented in a histogram, and a beta distribution is calculated from the mean and standard deviation of the observed values. A 97% quantile of the density function is used as a cutoff to asses orthology. The quantile has been identified by manual inspection of hundreds of histograms from real cases.

Roary and EDGAR are based on sequence alignment, however alternative strategies can be used for retrieving domain architecture between homologous genes [22] or for the detection of horizontal gene transfer [23], by exploiting alignment-free techniques.

We present PanDelos 1, a methodology to discover pan-genome content in phylogenetically distant organisms based on information theory and network analysis. It is parameter-free, the thresholds are automatically deduced from the context. PanDelos avoids sequence alignment by introducing a similarity measure based on *k*-mers multiplicity, rather than simple presence/absence of mers. The strength of the strategy is supported by a non-empirical choice of the best appropriate *k*-mer length. Moreover, the selection of minimum similarity for which two sequences are eligible to be homologs is inspired by the knowledge coming from read mapping in next-generation sequencing and sequence reconstruction processes. Reciprocal best hits in 1-vs-1 genome comparison, aimed at discovering orthologous genes, are used as a basis to infer thresholds for paralogs discovery. Homology relations are integrated into a global network and groups of homologous genes are extracted from it by applying a community detection algorithm. PanDelos outperforms in terms of running times and quality discovery contents the existing approaches, Roary and EDGAR, in real applications and in synthetic benchmarks, that represent fully trusted golden truth.

## Methods

**Fig. 1:**
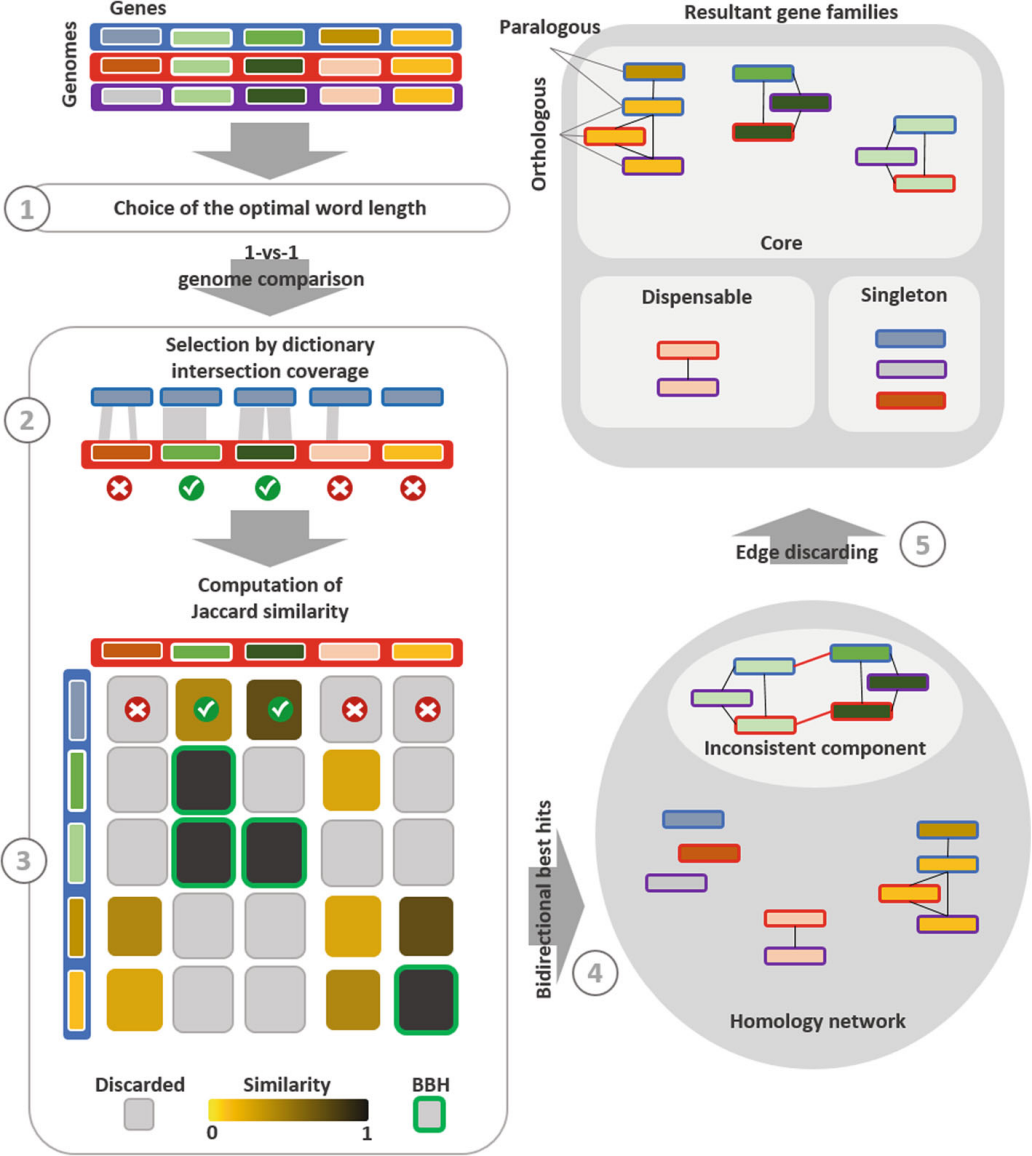
Overview of PanDelos Pan-genome computation of three genomes (represented as blue, red, and violet). Genomes are taken in input as list of genetic sequences (represented as colored rectangles). The homology detection schema is divided into 5 steps. PanDelos, at first, chooses an optimal word length that is used to compare dictionaries of genetic sequences. The 1-vs-1 genome comparisons are performed. An initial candidate gene pairs selection is obtained by applying a minimum percentage threshold on the dictionary intersection. Then, PanDelos computes generalized Jaccard similarities among genes (shown in the bottom left matrix). Only pairs of genes that passed the threshold applied on dictionary percentages are taken in consideration for the similarity computation. Pairs that did not pass the threshold are represented by gray tiles. Next, PanDelos computes bidirectional best hits (BBH), here represented with green borders. On the bottom right, a similarity network, made of reciprocal best hits is shown. Border colors represent the genomes to which genes belong. A final computational step discards edges in inconsistent components of the network and returns the final list of gene families. A component is inconsistent if it contains two genes belonging to the same genome that are not accounted as paralogs. A family may contains orthologous as well as paralogous genes, such as the yellow/brown ones. Families are finally classified as singletons, dispensable or core depending on their presence among genomes (borders of the rectangles represent the genomes the genes belong to)

The detection of gene homology performed by PanDelos is divided into 5 main steps that combine a candidate selection based on *k*-dictionaries, with a refinement procedure, developed by means of network analysis. Firstly, an optimal value of word length *k* is chosen according to properties of the input collection of genomes. Consequently, genes are compared and candidate homologous pairs are selected. The selection is firstly applied by setting a minimum amount of intersection between the *k*-dictionaries of two genes. Then, the generalized Jaccard similarity is used to measure the similarity between genes in order to extract bidirectional best hits. The extraction produces a homology network from which, at the end of a refinement procedure, gene families are retrieved. Figure 1 gives an overview of the overall schema.

In what follows, we first describe the details of PanDelos together with the engineering and extension of existing data structures that allows PanDelos to reach high performance and efficiency.

### Basic notation

A gene is represented as a string *s* over the amino acid alphabet *Γ*, *s*=*a*_1_*a*_2_…*a*_*h*_, with *a*_*i*_∈*Γ* for 1≤*i*≤*h*. The *k*-mers of *s* are the substrings of *s* having length *k*. A sequence *s*, having length |*s*|, contains |*s*|−*k*+1 occurrences of *k*-mers. A *k*-mer *w* may occurs several times within *s*. The number of times that *w* occurs in *s* is called the *multiplicity* of *w* in *s* and it is denoted by *c*_*s*_(*w*). The *k*-dictionary *D*_*k*_(*s*) of *s* is given by the set of all distinct *k*-mers occurring in *s*:

$$D_{k}(s) = { s[i..i+k] : 1 \leq i \leq |s| - k}, $$

where *s*[*i*..*i*+*k*] is the substring of *s* starting at position *i* and ending after *k* positions.

Given a population of *n* individual genomes, we denote by $\mathbb {G}^{i} = \{s_{1}, s_{2},\dots,s_{m} \}$ the set of genes of the *i*-th individual. The genetic length of $\mathbb {G}^{i}$ is given by the sum of the lengths of the genes in $\mathbb {G}^{i}$ and it is denoted by $\langle \mathbb {G}^{i} \rangle $. On the contrary, when whole DNA sequences are taken into account the genomic length of the *i*-th individual, |*G*^*i*^|, is given by the total length of the DNA sequence. In what follows, we use the term genome to indicate both a DNA sequence *G* and the corresponding set of genes $\mathbb {G}$. The context will suggest the intended appropriated meaning.

### Choosing an optimal word length for gene dictionary construction

A dictionary-based measure is highly sensitive to the length *k* of the words that compose the dictionary. In analyzing whole genome sequences, a crucial resolution is given by *k*=*log*_4_|*G*|, where 4 is the cardinality of the nucleotide alphabet [24, 25]. This value was proven to reveal structural laws that emerge from the maximum entropic difference between real genomes with random ones of the same length. In our case, genes are represented by the amino acid sequences of the proteins they encode, thus the alphabet to be considered is *Γ* rather than the 4-symbols nucleotide alphabet. Thus, we take into account the set of genetic sequences belonging to all the *n* input genomes by setting the value of the optimal word length *k* as:

$$k = {log}_{|\Gamma|} \sum\limits_{i=1}^{n} \langle \mathbb{G}^{i} \rangle. $$

### Selection of candidate gene pairs

Unfortunately, no theory exists to define a non-empirical threshold regarding the application of the Jaccard similarity in the context of gene comparison. Thus, a preliminary step filters pairs of gene candidate to be homologous. The intersection coverage of the dictionaries of two genes is used as a criterion of relational relevance between sequences. The criterion requires that the *k*-mers of *D*_*k*_(*s*∩*t*)=*D*_*k*_(*s*)∩*D*_*k*_(*t*) have to occur in *s* and *t* with a minimal percentage.

PanDelos creates a set *CH* of *candidate homologous genes* by computing, for each pair of genes *s* and *t*, $s \in \mathbb {G}^{i}$ and $t \in \mathbb {G}^{j}$, the percentage of *k*-mer occurrences of *s* that belong to $\widehat {D_{k}}(s, t)$. It is given by

$$p_{k}(s \rightarrow t) = \frac{{\sum\nolimits}_{w \in D_{k}(s, \cap t)} c_{s}(w)}{|s| - k + 1}. $$

PanDelos considers as homologous two genes *s*,*t* such that both *p*_*k*_(*s*→*t*) and *p*_*k*_(*t*→*s*) must overcome the minimum amount of 2/*k*.

The threshold 2/*k* is not empirically defined, but motivated by an argument that we will briefly outline. If we consider that from a sequence *s* we can extract at most |*s*|/*k* distinct non-overlapping *k*-mers, then we realize that, when the average multiplicity of *k*-mers in *s* is close to 1, this fraction is close to 1/*k* of the number of all *k*-mer occurrences of *s*. However, the lack of overlap denies any possibility of reconstructing of *s* from such a *k*-dictionary, because in this case there is no indication on how the different *k*-mers must be arranged to form *s*. Therefore, we assume that a minimum amount of overlap between consecutive *k*-mers extracted from *s* is given by doubling the above fraction 1/*k*. This argument suggests us to fix as 2/*k* the threshold of *p*_*k*_(*s*→*t*) and *p*_*k*_(*t*→*s*). In conclusion, *s* and *t* are considered homologous candidate genes if: *p*_*k*_(*s*→*t*)≥2/*k* and *p*_*k*_(*t*→*s*)≥2/*k*.

### Dictionary based gene sequence similarity detection

For each pair of genomes, $\mathbb {G}^{i}$ and $\mathbb {G}^{j}$, and for each candidate pair of genes (*s*,*t*), such that $s \in \mathbb {G}^{i}$ and $t \in \mathbb {G}^{j}$, PanDelos computes their sequences similarity by applying a generalized Jaccard similarity among the *k*-dictionaries. Note that, in the search for paralogous genes, *i* is equal to *j*.

Given two sequences, *s* and *t*, and *D*_*k*_(*s*∪*t*)=*D*_*k*_(*s*)∪*D*_*k*_(*t*) the union of their *k*-dictionaries, PanDelos uses the following generalized Jaccard similarity *J*_*k*_(*s*,*t*) on *k*-mer multiplicities:

$$J_{k}(s,t) = \frac{ {\sum\nolimits}_{w \in D_{k}(s \cup t)} min(c_{s}(w), c_{t}(w)) }{{\sum\nolimits}_{w \in D_{k}(s \cup t)} max(c_{s}(w), c_{t}(w))}. $$

It takes values in the interval [0,1]. It is independent of the lengths of the compared sequences and thus it is suitable for comparing sets of sequences having a wide range of lengths.

### Extraction of gene pairs by bidirectional similarity

In order to obtain the set *CH*_*O*_ of *orthologous candidate genes*, PanDelos computes bidirectional best hits (BBHs) on genes in *CH*.

Given a gene $s \in \mathbb {G}^{i}$, the set of best hits of *s* towards a genome $\mathbb {G}^{j}$ is given by:

$$BH\left(s, \mathbb{G}^{j}\right) = \left\{ t \in \mathbb{G}^{j} : J_{k}(s,t) = \underset{v \in \mathbb{G}^{j}}{\text{max} } J_{k}(s,v) \right\}. $$

The set of bidirectional best hits of *s* towards $\mathbb {G}^{j}$ is given by:

$$BBH\left(s, \mathbb{G}^{j}\right) \,=\, \left\{ t \in \mathbb{G}^{j} : t \in \!BH\left(s,\mathbb{G}^{j}\right) \text{and}\ s \in BH\left(t, \mathbb{G}^{i}\right) \right\} $$

Only genes involved in at least one BBH are kept in *CH*_*O*_.

The BBH strategy is commonly used in pan-genomic analyses, however, it may capture sequences having low similarity. This behavior especially arises with singleton sequences. Two unrelated singletons of the two genomes may form a BBH simply because no orthologs exist and they are reciprocally the best match. PanDelos avoids these cases by performing the BBH strategy only on genes in *CH*, i.e. on candidate genes ‘similar enough’.

In order to obtain the set *CH*_*P*_ of *paralogous candidate genes*, at the end of every 1-vs-1 genome comparison, the minimum score of BBH orthologous candidate genes is used to infer new paralogous. Recalling that PanDelos has compared each genome to it self, the intra-genome BBH with a score equal to or greater than the minimum inter-genomes BBH score (orthology score) are accounted as paralogous. This rule states that the score accounting for orthologous sequences can be used as threshold to define two genes as paralogous because their similarity is strong at least as the minimum trusted similarity between orthologs.

### Gene family detection by network coherence refinement

PanDelos constructs an undirected weighted network from homology information, where each vertex is labelled with a pair $(s, \mathbb {G}_{i})$ formed by a candidate gene and the genome to which it belongs, and where an edge connects two vertices if they are in *CH*_*O*_ or *CH*_*P*_. The edge weights are the scores computed applying the generalized Jaccard similarity on candidate genes.

The network may be formed by several connected components which are the starting homologous candidate gene families. A connected component is defined *inconsistent* if it contains two genes belonging to the same genome that are not accounted as paralogs, namely which are not connected by an edge. The inconsistency is resolved by recursively splitting the component into subgroups until a set of consistent subgroups is reached. PanDelos uses the Girvan-Newman algorithm for community detection which calculates the betweenness centrality along the components and progressively removes the edge with the highest centrality [26]. PanDelos normalizes the edge weights by means of the maximum weight present in each connected components.

The resulting pan-genomic structure is given by the final set of consistent connected components plus singleton genes, i.e. the singleton vertices in the network. Components containing genes of all genomes represent the *core* of the pan-genome. The other components contain the *dispensable* genes.

### Data structures engineering for fast similarity computation

#### Limitations of enhanced suffix arrays for pan-genome computations

**Fig. 2:**
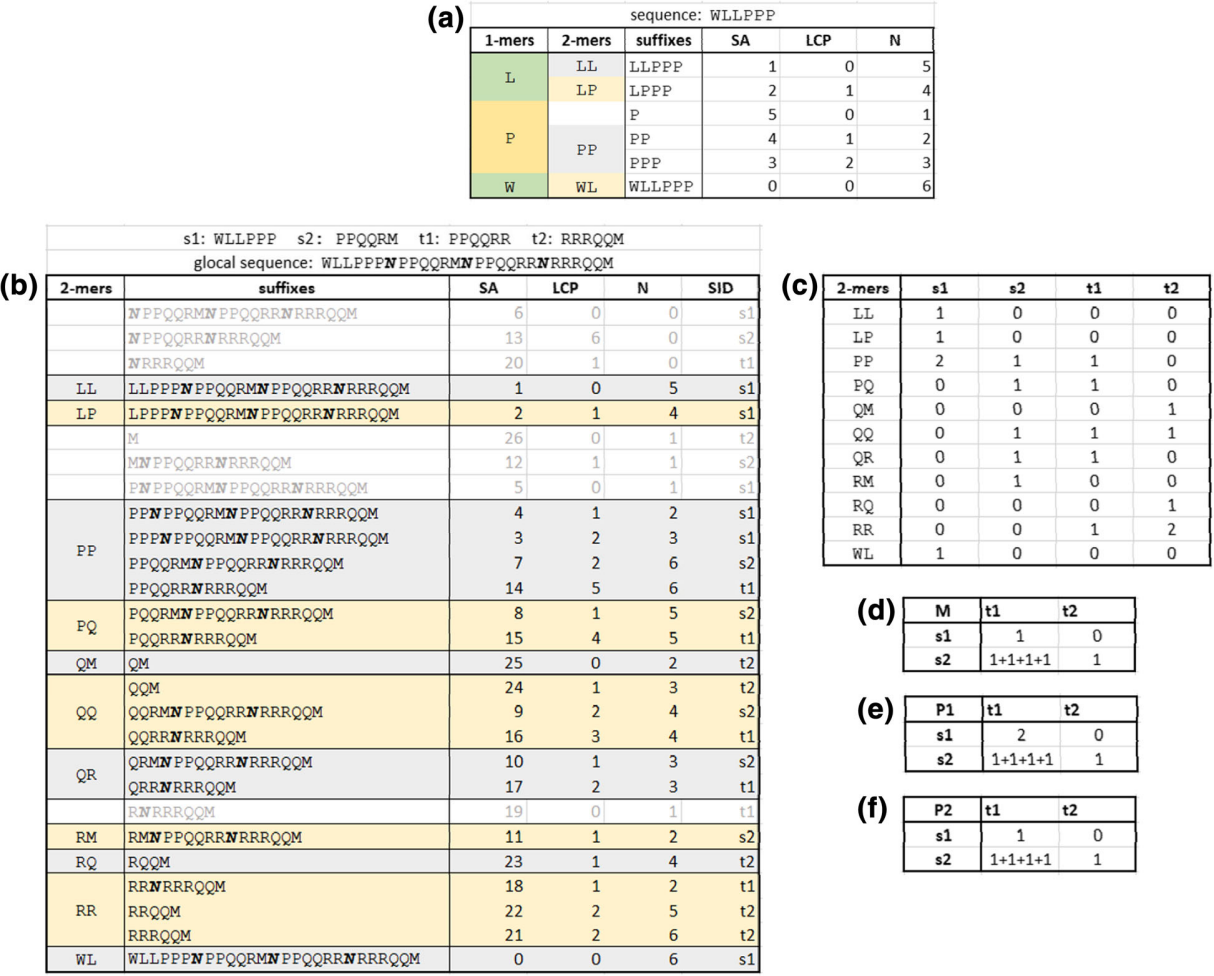
Examples of indexing structures. In the top-side of the image **a**, an example of the indexing structure ESA+N is shown for the string *WLLPPP*. The string is indexed by lexicographically sorting its suffixes. The array *SA*, *LCP* and *N* are computed according to the ordering. The indexing structure is composed by the three arrays, and the other columns shown on the image are virtually extracted. The *SA* array stores star positions of suffixes and it is used to keep trace of the lexicographic ordering. Values along the *LCP* and *N* arrays are used to identify intervals that correspond to specific k-mers [30]. The 1-mer *L* is identified by an interval that covers the first two positions of the structure, while the 1-mer *P* covers three positions and the 1-mer *W* cover one positions. Thus, the multiplicity of *L*, *P* and *W* are respectively 2, 3, and 1. 2-mers intervals are shown in the second columns,from the left. Note that the third position is not cover when 2-mer intervals are extracted because it can not identify the start of any 2-mer. The second section of the image **b**, **c**, **d**, **e**, **f**, show the extension the indexing structure in order to manage set of strings. Four input strings, *s*1, *s*2, *t*1 and *t*2 are indexed. Firstly, a global string is built by concatenating the four strings and by putting a special symbol (represented as **N**) on the concatenation joints. Then, similarly to the single string case, suffixes of the global sequences are sorted in lexicographic order. The sorting procedure defines the content of the *SA* array and *LCP*, *N* and *SID* arrays are computed in accordance with it. The *SID* array informs for each suffix the sequences from which it originates. The indexing structure helps in extracting the information, namely the multiplicities of 2-mers in every sequence, that is ideally represented in the matrix **b**. The illustrations **d**, **e** and **f** show the final values that the matrices *M*, *P*_1_ and *P*−1 take after every 2-mer of the global sequence have been taken into account

Given a string *s*, a suffix array (SA) [27] reports the lexicographically ordered suffixes of *s* equipped with their start position in *s*. Substring search by means of SA can be sped up by performing binary searches. An enhanced suffix array (ESA) [28] is a combination of SA with the LCP (Longest Common Prefix) array giving the length of the longest common prefix of a suffix with that one lexicographically preceding it. An ESA allows for efficient recovery of the *k*-mers multiplicities [29]. The values of the LCP array define contiguous regions of the ESA array, called LCP-intervals, which identify all the occurrences of *k*-mers. Additionally, an array of length N reports for each suffix the distance from its start to the first forward occurrence of a *N* symbol [30]. The *N* is used to represent positions in *s* that must be discarded in dictionary operation. The ESA structure performs *k*-mer enumeration in linear time by just doubling the memory requirement of simple SA. Since each *k*-mer must be checked for *N* inclusion, this verification increases the time complexity by a factor of *k*. However, with the additional N array, the complexity remains linear. Figure 2a gives an example of ESA+N structure that has been built for the string *WLLPPP*, and illustrates LCP intervals of 1-mers and 2-mers of the string.

Methodologies for efficient similarity calculation in sets of strings have been developed by means of suffix trees [31]. This type of data structure inspired the develop of suffix arrays as a memory efficient implementation that does not increase time requirements. However, enhanced suffix arrays are useful for single reference string analysis, but result less suitable to be applied to sets of strings.

Given two sequences, *s* and *t*, the comparison of their *k*-dictionaries can be performed by listing the *k*-mers of *s* and searching for them in *t*. Taking into account an ESA+N structure, the *k*-mers listing is performed in |*s*| time, and the search of each *k*-mer in *t* takes $\mathcal {O}(k \cdot log(|t|))$ time. Since at most |*s*| distinct *k*-mers are in *s*, the overall time is $\mathcal {O}(|s| \cdot k \cdot log(|t|))$.

The search described above takes into account only *D*_*k*_(*s*), but *t* may contain *k*-mers not listed in the dictionary of *s*, thus the time requirement is doubled because *D*_*k*_(*t*) must also be scanned. In 1-vs-1 genome comparison, the process must be repeated for every pair of genes belonging to the two genomes, resulting in a highly expensive procedure. In the next section, we address a way to efficiently improve the procedure.

#### PanDelos data structure engineering

We extend the ESA+N structure [30] in order to speed up the comparison of *k*-dictionaries when multiple sequences are taken into account. The goal is to compute the generalized Jaccard similarities between a set of sequences simultaneously.

The generalized Jaccard similarity between *s* and *t* can be expressed as:

$$J_{k}(s,t) = \frac{a}{b + c}, $$

where *a* is the sum of the minimum multiplicities of *k*-mers shared by the two sequences, *b* is the sum of the maximum multiplicities of the shared *k*-mers, and *c* is the sum of the multiplicities of *k*-mers appearing only in one of the two sequences.

Given *a* and *b* for every pair of sequences, then *c* is obtained as

$$c = (|s| + |t| - 2k + 2) - (a + b), $$

where (|*s*|+|*t*|−2*k*+2) is the sum of multiplicity of all *k*-mers in *s* and *t*. Therefore it can be rewritten as:

$$J_{k}(s,t) = \frac{a}{|s| + |t| - 2k + 2 - a}. $$

Given two genomes, $\mathbb {G}^{1}=\{s_{1}, \dots, s_{n}\}$ and $\mathbb {G}^{2}=\{t_{1}, \dots, t_{m}\}$, genes are concatenate in a single global sequence *s*_1_·*N*·⋯·*N*·*s*_*n*_·*N*·*t*_1_·*N*·⋯·*N*·*t*_*m*_. An ESA+N structure is built, and the concatenation by *N* symbols ensures that extracted *k*-mers do not cross between gene sequences. The data structure is extended with a further array, called *SID*. Given an LCP-interval, that represents a specific *k*-mer, the content of the corresponding interval in the *SID* array reports the identifiers of the sequences in which the *k*-mer is present. Moreover, the number of time a sequence identifier is repeated within the interval corresponds to the multiplicity of the *k*-mer within the specific sequence.

For each pair of sequences involved in the interval, we compute the sum of minima (the *a* term in the generalized Jaccard formula) by computing the partial of such sums and storing them in a matrix *M*

$$M[i,j] = \sum\limits_{w \in D_{k}\left(s_{i} \cup t_{j}\right)} min\left(c_{s_{i}}(w), c_{t_{j}}(w)\right), $$

for 1≤*i*≤*n* and 1≤*j*≤*m*.

Once the matrix is filled, the similarities are finally computed by the formula:

$$J\left(s_{i},t_{j}\right) = \frac{ M[i,j] }{ \left|s_{i}\right| + \left|t_{j}\right| -2k +2 - M[i,j]}. $$

In this way, we avoid comparisons between sequences that do no share any *k*-mers, and eliminate the logarithmic factor of searching *k*-mers into multiple suffix arrays, one for each gene sequence.

Similarly, two additional matrices are stored in order to calculate candidate homologous genes: *P*_1_[*i*,*j*] reporting the percentage of multiplicities of *k*-mers in *s*_*i*_shared with *t*_*j*_, and *P*_2_[*i*,*j*] storing the vice versa:

$$P_{1}[i,j] = \sum\limits_{w \in D_{k}(s_{i} \cap t_{j}) }\frac{c_{s_{i}}(w)}{|s|-k+1} $$

$$P_{2}[i,j] = \sum\limits_{w \in D_{k}(s_{i} \cap t_{j})} \frac{c_{t_{j}}(w)}{|t|-k+1}. $$

Figure 2 reports an example for four sequences that are concatenated into a single one. Then 2-mers, and their associated sequences identifiers, are retrieved from the data structure. In the example, two genomes are compared. The first genome contains the sequences *s*_1_ and *s*_2_, and the second genome contains the sequences *t*_1_ and *t*_2_. The word length 2 is chosen as best *k* value, thus sequences are compared by means of the multiplicities of the 2-mers they contain. Ideally, the matrix **(c)** has to be computed in order to calculate the Jaccard similarity between the sequences. For higher values of *k*, the storage and update of such matrix may require high computational efforts, thus its rows are computed *on the fly* by identifying k-mer intervals along the indexing structure and by iterating them. A linear iteration over the structure lists the 2-mers, together with the number of times they appear within each original sequence. During the iteration the three matrices *M*, *P*_1_ and *P*_2_, in Fig. 2d, e and f, are updated. After that every 2-mer have been iterated, Jaccard similarity are computed by means of the *M* matrix, while coverage percentage are computed by means of the *P*_1_ and *P*_2_ matrices.

## Results

PanDelos has been compared to Roary and EDGAR. Roary is a stand-alone computational tool written in Perl. It runs under Linux systems and is takes as input genomic data in GFF format. We used the tool with its default parameter settings, except for the experiments regarding the *Mycoplasma* genus where we performed parameter tuning to improve its performance. EDGAR is a web based tool, it gives precomputed analyses performed on individuals grouped by living species. PanDelos is a pipeline composed of Java and Python modules and takes in input genomic data in GFF format. Tests were run on a machine equipped with an Intel Core i7-5960X CPU and 64 Gb of RAM on top of which an Ubuntu 16.04 64 bit Linux OS is installed.

Several notions of phylogenetic distance have been defined in the literature. Each distance captures a specific aspect of genome evolution. Here, we refer to a distance [32] that is widely used to infer phylogenetic trees of bacterial populations [33]. The measure computes the cosine similarity between the composition vectors of the proteomes of the compared genomes. The distance reaches a minimum of 0 for genomes having the same composition, and a maximum of 1 for completely unrelated proteomes.

### Comparisons on real collections of genomes

We compared PanDelos, EGDAR and Roary on four real cases. We used two collections of genomes originally used to evaluate the performances of Roary and EDGAR, i.e. 7 isolates of the *Typhi* serotype of the *Salmonella enterica* species which is known to have very closely related genomes, and 14 isolates of the *Xanthomonas campestris* species. The *Typhi* serotype and the *Xanthomonas* genus has been used as reference case to study performances respectively of Roary and EDGAR. We further selected, from EDGAR available datasets, 10 isolates of *Escherichia coli* species, and 64 isolates of *Mycoplasma* genus. These two collections show opposite properties for what concerns phylogenetic distances, in fact, the primer is a group of closely related genomes and the latter represents a collection of highly distant sequences. The identifiers of the selected isolates are reported in Additional file 1: Tables S1–S4. Their properties, summarized in Additional file 1: Figures S1–S4, show changes in the number of sequences of the genomes. An high variability in genetic sequence lengths is also reported (from 13 to thousands of amino acids).

Table 1 reports the average phylogenetic distances within the analyzed real populations. The *Escherichia coli* dataset has the most similar genomes, in fact their phylogenetic distances reach the lowest values. The population with the higher variability is given by the *Mycoplasma* dataset. Details regarding phylogenetic distances of these datasets are show in Additional file 1: Figures S5–S8.

**Table 1:** Phylogenetic distances (average and standard deviation) for the four real datasets

Species	Distance
*Escherichia coli*	0.28 (0.13)
*Salmonella enterica*	0.37 (0.34)
*Xanthomonas campestris*	0.69 (0.25)
*Mycoplasma*	0.92 (0.21)

The three tools show a similar performance on the 7 closely related isolate of *Salmonella enterica*, whereas Roary showed low performances on *Xanthomonas campestris* collection (see Tables 2 and 3). For each tool, the number of gene families shared among genomes that compared tools have found is shown. Singletons appear in only one genome, core genes are shared among all the 7 genomes, and the remaining accessory genes are shared from 2 to 6 genomes. On the contrary, the comparison related to the *Xanthomonas campestris* collection showed a low performance of Roary (see Table 3). In fact, while PanDelos and EDGAR found circa 9k gene families, Roary reported more than 17k groups. Roary found a double amount of singletons and groups shared among a low number of genomes. Notably, PanDelos and EDGAR discovered the presumably correct pan-genome content having a high number of singletons and core genes. Similar results are reported for the *Escherichia coli* (see Table 4) collection. However, as for the *Xanthomonas campestris* collection, Roary found a large number of families in sets composed by a low number of genomes. The percentage of core genes, w.r.t. the total aggregated families, computed by PanDelos was 37%, while Roary reached a percentage equal to 31%. In *Mycoplasma* isolates, PanDelos found 22 core genes while EDGAR only 14 (see Table 5). Among those 14 genes, only one was absent on the list of 22 core genes given by PanDelos. PanDelos and EDGAR found a total of 13,181 and 12,344 families, respectively, thus the core percentages are less than 1% (0.16% and 0.17%). Roary, launched with default parameters, did not detect core genes. It found dispensable families shared among at most 12 genomes and did not discover genes with higher sharing. We decided to reduce the Roary threshold on the BLAST score, which is by default equal to 95%, until Roary reported core genes. With an identity threshold set to 65%, Roary found only 2 core genes. A detailed description of the core genes found by the three approaches in given in Additional file 1: Table S5. PanDelos and EDGAR are in accordance for 10 core genes. The results obtained for all the four collections are graphically summarized in Additional file 1: Figure S9.

**Table 2:** Number of genomes per gene family in 7 serotype *Typhi* of the *Salmonella enterica* species

Genome count	PanDelos	EDGAR	Roary
1	241	219	236
2	74	72	87
3	27	28	35
4	93	42	108
5	213	246	213
6	469	491	464
7	3748	3749	3751
Total	4865	4847	4894

The table reports the count of gene families found in a given amount (from 1 to 7) of genomes, for each of the tested algorithms. Families found in only 1 genome are the singletons, whereas families found in all 7 genomes are the core families. The whole dataset consists of 31,311 gene sequences (CDS) that were clustered in more then 4800 gene families by the three approaches

**Table 3:** Number of genomes per gene family in 14 *Xanthomonas campestris* species

Genome count	PanDelos	EDGAR	Roary
1	3050	2572	7143
2	743	854	1864
3	797	873	2112
4	585	600	3811
5	233	249	1086
6	159	201	86
7	110	111	40
8	128	143	104
9	400	431	797
10	196	222	12
11	107	98	13
12	203	181	54
13	715	630	642
14	1742	1829	50
Total	9168	8994	17814

The table reports the count of gene families found in a given amount (from 1 to 14) of genomes, for each of the tested algorithms. The dataset consists of a total of 56,759 input gene sequences

**Table 4:** Number of genomes per gene family in 10 *Escherichia coli* isolates

Genome count	PanDelos	EDGAR	Roary
1	1819	1593	2589
2	740	781	1083
3	916	990	1270
4	463	527	523
5	287	301	265
6	322	332	290
7	201	223	172
8	228	224	145
9	354	338	312
10	3075	3084	2951
Total	8405	8443	9600

The dataset consists of a total of 48,980 input gene sequences

**Table 5:** Number of genomes per gene family in 64 *Mycoplasma* genus

Genome count	PanDelos	EDGAR	Roary	Roary-65
1-10	12,218	11,180	21,140	15,240
11-20	676	825	604	737
21-30	156	166	0	59
31-40	31	54	0	4
41-50	38	51	0	6
51-60	27	40	0	5
61-64	35	28	0	5
Total	13,181	12,344	21,744	16,056

The table reports the count of gene families found in a given amount (from 1 to 64) of genomes, for each of the tested algorithms. The dataset consists of a total of 47,385 input gene sequences

### Comparisons on collections of synthetic genomes

Since in real data we don’t know the exact phylogeny, we created a synthetic benchmark simulating genomes evolution.

The generated population can be represented as an *n*-ary tree, where the root is the common ancestor genome. Leaves of the tree are genomes without progeny. Starting from an existing genome, we generated descendants by vertical transmission (copy of parent genes), loss of parent genetic material or addition of new genetic material (in order to simulate horizontal transfer). Synthetic generation of gene families is a studied problem in literature [34]. However, few studies have proposed the development of a methodology to simulate genome evolution in a pan-genome context. The IGM model [35] simulates vertical and horizontal transmission but the actual implementation is able to create and evolve genes having almost the same lengths, that is an unfeasible behavior considering the real variability in gene length. The SimBAC approach [36] simulates variations at the genomic level that can occur during bacterial evolution but it does not keep trace of gene transmission. Thus, the real homology relationships are lost. For these reasons, we decided to implement an in-house procedure, in order to simulate bacterial evolution, that is inspired by existing approaches. The procedure traces homology relationships and generates synthetic collections that show properties similar to real bacterial populations. The procedure is briefly described above.

From a parent gene set, 0.1*%* of genes are removed and 1% totally new genes are added. The 80% of the transmitted genes were varied by adding, removing or changing a given percentage of amino acids. Finally, the 0.01*%* of the genes were duplicated. We generated 4 populations of 2000 individuals from 2 real *Mycoplasma* genomes by applying two different variation percentages, 0.5*%* and 1%. From each population, we extracted the 50 individuals closest to the ancestor genome, referred to as *roots*, and 50 peripheral individuals (leaves of the n-ary tree), referred to as *leaves*.

Additional file 1: Figure S10 shows phylogenetic relationships of the four 2k-individuals populations. Structural properties of the phylogenetic tree of one of the populations are reported in Additional file 1: Table S6. The number of total individuals, the number of peripheral genomes, and the average number of descendants are shown for each depth of the tree. The generated populations show compositional properties, namely number of genes per genomes, variation of gene lengths within each genome and pan-genomic trends that are similar to real collections (see Additional file 1: Figure S11). The realistic composition and trends are also maintained in the 50-individuals sub-populations (see Additional file 1: Figure S12). Table 6 reports average phylogenetic distances within the extracted sub-populations and further details are given in Additional file 1: Figures S13–S20. The synthetic sub-populations show realistic distances. The *roots* extracted from populations generated with 0.5*%* locus variation percentage seem to show unrealistic average distances, however, the detailed information shows genomic distances similar to the *Escherichia coli* collection.

**Table 6:** Phylogenetic distances (average and standard deviation) for the four extracted synthetic sub-populations

G37 (*Mycoplasma genitalium*)		
	Variation perc.	
Extr. type	0.5%	1%
Roots	0.17 (0.6)	0.24 (0.07)
Leaves	0.55 (0.17)	0.68 (0.17)
M129 (*Mycoplasma pneumoniae*)		
	Variation perc.	
Extr. type	0.5%	1%
Roots	0.15 (0.05)	0.22 (0.07)
Leaves	0.55 (0.14)	0.64 (0.19)

We used the synthetic dataset as a golden truth to compare PanDelos, EDGAR and Roary on quality of retrieved families. We evaluated the performances of the methodologies by comparing the set of homology relationships that they extract from the input genomes and how such relationships infer the pan-genomic distribution.We measured the number of true positive (TP) relationships retrieved by each approach, the correct homologies; the number of false positive (FP) relationships, i.e. the wrong reported homology relationships; the number of true negative relationships (TN), i.e. the correct discarded homology relations; the number of false negative relationships (FN), i.e. the links that are not extracted by the approach but that are present in the synthetic data. Then we combine the above measures into an f-measure which informs about the accuracy of the results. The measure reaches the best value at 1 and the worst at 0.

Table 7 shows that PanDelos and EDGAR keep an high amount of true positives, that is also reported for Roary on *roots* collections. Roary significantly decreases TP in *leaves* collections, and this behavior is directly linked to an increase in false negatives. PanDelos and EDGAR are mostly not affected by false positives, while Roary decreasing performance follows the increase in the number of input sequences. The total number of possible relationships reaches the order of billions of links when all the input sequences can be linked to each other. However, PanDelos and EDGAR show a good performance in discarding most of unfeasible relationships (TN values). Both algorithms have f-measure values closed to 1 for every collection, but PanDelos shows higher values. Roary shows very low performances in *leaves* datasets. This behavior is also reflected in the CDiff value, which measures the number of gene families that have been erroneously split or merged by the tools w.r.t. the golden truth. Ideally, a gene family is a connected component in the homology network formed as a clique, namely every possible edge between the vertices of the component are present. A discovery methodology may miss some of the edges in a component, but without losing the whole connectivity. On the contrary, high amounts of missing edges may split components, and wrongly assigned links may merge multiple components. CDiff values reported for PanDelos are mostly linked to phylogenetic distances, in fact, low values are reported for collections of highly similar genomes, the *roots*, and higher values are expressed for datasets of more distant genomes, the *leaves*. A similar trend is observed for CDiff values of EDGAR, however, the methodology is affected by higher values compared to PanDelos.

**Table 7:** Performances of PanDelos, EDGAR and Roary on the synthetic datasets

			TP	FP	FN	TN	f-measure	CDiff
PanDelos								
G37	0.5%	Roots	1,263,632	0	2324	1,386,060,388	0.9991	1
M129	0.5%	Roots	1,689,082	0	4060	2,460,563,516	0.9988	2
G37	1%	Roots	1,259,344	0	5310	1,385,219,936	0.9979	0
M129	1%	Roots	1,689,682	0	3024	2,467,589,288	0.9991	0
G37	0.5%	Leaves	1,278,188	0	25,374	1,756,053,000	0.9902	24
M129	0.5%	Leaves	1,695,228	0	47,376	3,086,359,452	0.9862	57
G37	1%	Leaves	1,270,658	0	45,042	1,735,332,524	0.9826	64
M129	1%	Leaves	1,773,110	196	57,320	3,144,153,456	0.9840	210
EDGAR								
G37	0.5%	Roots	1,258,382	0	7574	1,386,060,388	0.9970	34
M129	0.5%	Roots	1,663,846	0	29,296	2,460,563,516	0.9913	139
G37	1%	Roots	1,253,564	0	11,090	1,385,219,936	0.9956	48
M129	1%	Roots	1,665,186	0	27,520	2,467,589,288	0.9918	132
G37	0.5%	Leaves	1,269,670	0	33,892	1,756,053,000	0.9868	154
M129	0.5%	Leaves	1,671,400	0	71,204	3,086,359,452	0.9791	319
G37	1%	Leaves	1,269,724	0	45,976	1,735,332,524	0.9822	197
M129	1%	Leaves	1,753,318	98	77,112	3,144,153,554	0.9785	267
Roary								
G37	0.5%	Roots	1,212,344	0	53,612	1,386,060,388	0.9784	179
M129	0.5%	Roots	1,598,840	856	94,302	2,460,562,660	0.9711	247
G37	1%	Roots	1,166,946	0	97,708	1,385,219,936	0.9598	383
M129	1%	Roots	1,541,422	1244	151,284	2,467,588,044	0.9529	537
G37	0.5%	Leaves	348,356	112	955,206	1,756,052,888	0.4217	3520
M129	0.5%	Leaves	423,836	154	1,318,768	3,086,359,298	0.3912	5619
G37	1%	Leaves	97,710	24	1,217,990	1,735,332,500	0.1383	6302
M129	1%	Leaves	468,466	64	1,361,964	3,144,153,588	0.4075	4674

**Fig. 3:**
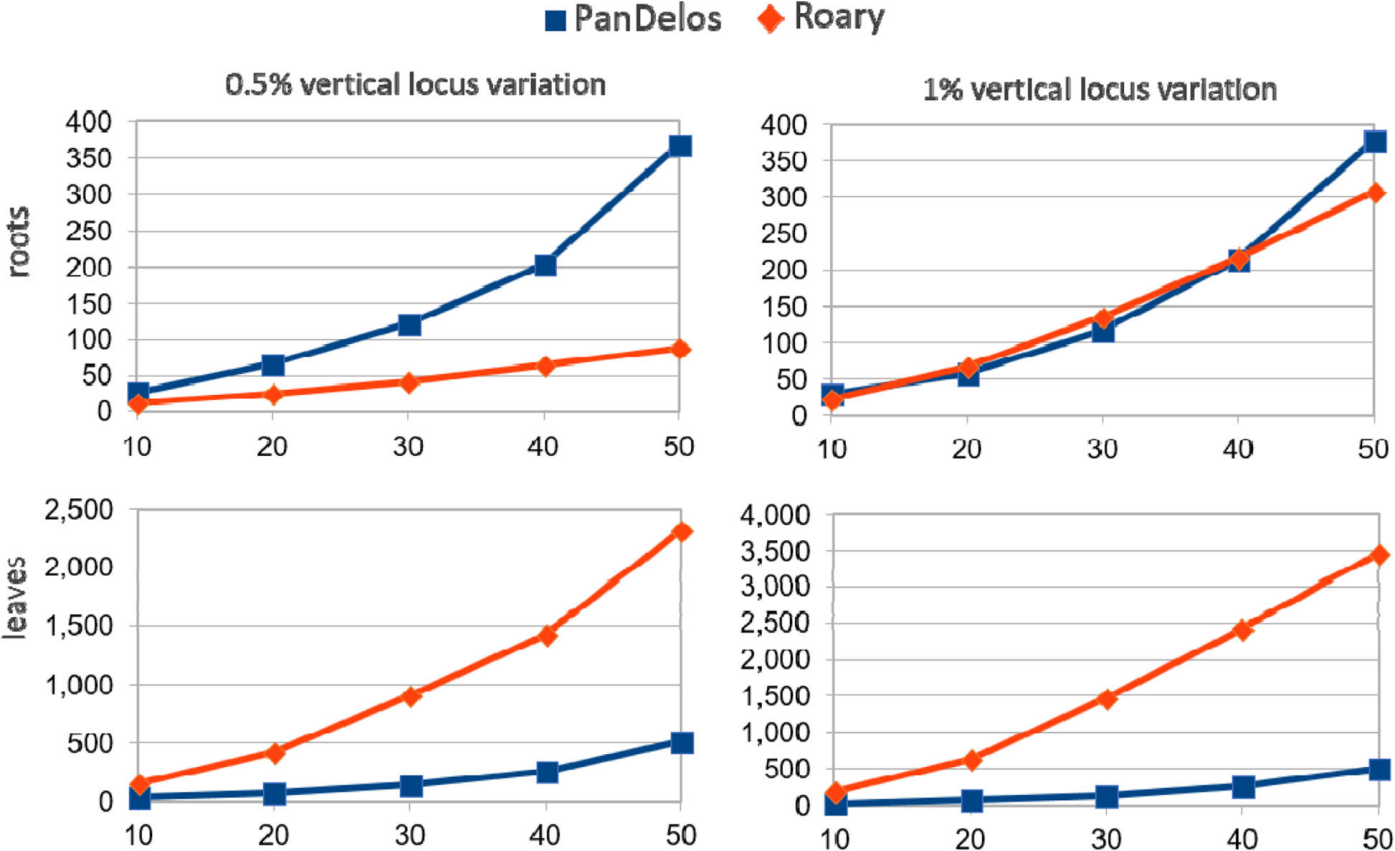
Execution times of PanDelos and Roary over the four synthetic datasets extracted form the two populations generated from the *Mycoplasma genitalium* G37 genome. Time requirements have been measured by taking into account five different amounts of analyzed genomes, from 10 to 50. Execution times are reported in seconds

Finally, we evaluated the execution times of PanDelos and Roary over synthetic data. Figure 3 shows time costs of the two methodologies on varying the number of analyzed genomes, from 10 to 50. Times were recorded for the four 2k-individuals datasets extracted from the populations generated starting from the *Mycoplasma genitalium* G37 genome. Roary outperforms PanDelos on the *roots* dataset generated with a 0.5*%* locus variation percentage that is the collection with the lowest, and probably unrealistic, average phylogenetic distance (see also Table 6). The two approaches show comparable performances on the *roots* dataset obtained with 1% locus variation. However, the 0.24 average distance of this collection is lower than the averages computed on real datasets (for which the minimum is 0.28 of the *Escherichia coli* collection, see Tables 1 and 6). PanDelos clearly outperforms Roary on *leaves* datasets (the ones that show average distances similar to real cases). Moreover, the performance of PanDelos is shown to be not affected by phylogenetic distances, but it is only dependent on the number of input genomes. This trend is in contrast with the performance of Roary that is affected by the number of input genomes and also by phylogenetic distance. In fact, for both datasets, the running time of PanDelos has a stable increase of 15x (from 32 to 507 s on 0.5*%* variation, and from 39 to 509 s on 1% variation). On the contrary, Roary has an increase of 14x (from 159 to 2320 s) on the 0.5*%* variation dataset, and 17x (from 202 to 3448 s) on the 1% variation dataset. Similar results were obtained by running the two tools on the synthetic datasets generated from the *Mycoplasma pneumoniae* M129 genome (see Additional file 1: Figure S21).

## Discussion

For what concerns collections regarding real cases, in general, Roary performs similarly to the two other methodologies on populations with low phylogenetic distances, namely *Salmonella enterica* and *Escherichia coli*, but it is quiet different on the other dataset by reporting a higher number of singletons and a lower amount of core families. On the contrary, PanDelos and EDGAR show coherent trends and the homology detection of both approaches can be considered realistic. However, PanDelos was able to detect more core genes in the collections having the highest and most variable phylogenetic distances.

Regarding synthetic benchmarks, the low performance of EDGAR, mainly expressed by CDiff values higher than PanDelos, may be linked to the high amount of false negative homology relations that cause the break of gene families into subgroups. This result agrees with the behaviors of PanDelos and EDGAR obtained on the collection of 64 real *Mycoplasma* individuals.

## Conclusions

We presented PanDelos, a methodology for discovering pan-genome contents of closely related and phylogenetic distant genomes. The advantages of the approach are the absence of user-defined parameters, a similarity measure based on dictionaries, and the choice of the optimal dictionaries by applying theoretical concepts emerging from informational analysis of genomes [24]. PanDelos dominates the intrinsic complexity of phylogenetic distances among input genomes by searching for gene communities over a global normalized homology network. Finally, PanDelos extends the suffix array data structure for efficiently computing the similarity between sets of sequences. Comparisons in real and synthetic cases have demonstrated the outperforming of PanDelos on the existing methods Roary and EDGAR.

## Additional file

Additional file 1 Supplementary materials of PanDelos: a dictionary-based method for pan-genome content discovery. (PDF 5543 kb)

## Abbreviations

BH: Best hit
BBH: Bidirectional best hit
ESA: Enhanced suffix array
LCP: Longest common prefix
SA: Suffix array

## Footnotes

1 Delos is the core island of the Cyclades archipelago.

## References

[1] Vernikos G, Medini D, Riley DR, Tettelin H (2015). Ten years of pan-genome analyses. Curr Opin Microbiol.

[2] Medini D, Donati C, Tettelin H, Masignani V, Rappuoli R (2005). The microbial pan-genome. Curr Opin Genet Dev.

[3] Tettelin H, Riley D, Cattuto C, Medini D (2008). Comparative genomics: the bacterial pan-genome. Curr Opin Microbiol.

[4] Holt KE, Parkhill J, Mazzoni CJ, Roumagnac P, Weill F-X, Goodhead I, Rance R, Baker S, Maskell DJ, Wain J (2008). High-throughput sequencing provides insights into genome variation and evolution in salmonella typhi. Nat Genet.

[5] Earle SG, Wu C-H, Charlesworth J, Stoesser N, Gordon NC, Walker TM, Spencer CC, Iqbal Z, Clifton DA, Hopkins KL (2016). Identifying lineage effects when controlling for population structure improves power in bacterial association studies. Nat Microbiol.

[6] Serruto D, Serino L, Masignani V, Pizza M (2009). Genome-based approaches to develop vaccines against bacterial pathogens. Vaccine.

[7] Muzzi A, Masignani V, Rappuoli R (2007). The pan-genome: towards a knowledge-based discovery of novel targets for vaccines and antibacterials. Drug Discov Today.

[8] Zhang Y, Sievert SM (2014). Pan-genome analyses identify lineage-and niche-specific markers of evolution and adaptation in epsilonproteobacteria. Front Microbiol.

[9] D’Auria G, Jiménez-Hernández N, Peris-Bondia F, Moya A, Latorre A (2010). Legionella pneumophila pangenome reveals strain-specific virulence factors. BMC Genomics.

[10] Brittnacher MJ, Fong C, Hayden H, Jacobs M, Radey M, Rohmer L (2011). Pgat: a multistrain analysis resource for microbial genomes. Bioinformatics.

[11] Contreras-Moreira B, Vinuesa P (2013). Get_homologues, a versatile software package for scalable and robust microbial pangenome analysis. Appl Environ Microbiol.

[12] Benedict MN, Henriksen JR, Metcalf WW, Whitaker RJ, Price ND (2014). Itep: an integrated toolkit for exploration of microbial pan-genomes. BMC Genomics.

[13] Chaudhari NM, Gupta VK, Dutta C. Bpga-an ultra-fast pan-genome analysis pipeline. Sci Rep. 2016; 6. 10.1038/srep24373 PMC4829868 27071527

[14] Nguyen N, Hickey G, Zerbino DR, Raney B, Earl D, Armstrong J, Kent WJ, Haussler D, Paten B (2015). Building a pan-genome reference for a population. J Comput Biol.

[15] Page AJ, Cummins CA, Hunt M, Wong VK, Reuter S, Holden MT, Fookes M, Falush D, Keane JA, Parkhill J (2015). Roary: rapid large-scale prokaryote pan genome analysis. Bioinformatics.

[16] Blom J, Kreis J, Spänig S, Juhre T, Bertelli C, Ernst C, Goesmann A (2016). Edgar 2.0: an enhanced software platform for comparative gene content analyses. Nucleic Acids Res.

[17] Rasko DA, Myers GS, Ravel J (2005). Visualization of comparative genomic analyses by blast score ratio. BMC Bioinformatics.

[18] Sahl JW, Caporaso JG, Rasko DA, Keim P (2014). The large-scale blast score ratio (ls-bsr) pipeline: a method to rapidly compare genetic content between bacterial genomes. PeerJ.

[19] Li W, Jaroszewski L, Godzik A (2001). Clustering of highly homologous sequences to reduce the size of large protein databases. Bioinformatics.

[20] Altschul SF, Gish W, Miller W, Myers EW, Lipman DJ (1990). Basic local alignment search tool. J Mol Biol.

[21] Enright AJ, Van Dongen S, Ouzounis CA (2002). An efficient algorithm for large-scale detection of protein families. Nucleic Acids Res.

[22] Syamaladevi DP, Joshi A, Sowdhamini R (2013). An alignment-free domain architecture similarity search (adass) algorithm for inferring homology between multi-domain proteins. Bioinformation.

[23] Cong Y, Chan Y-b, Ragan MA (2016). A novel alignment-free method for detection of lateral genetic transfer based on tf-idf. Sci Rep.

[24] Bonnici V, Manca V (2016). Informational laws of genome structures. Scientific reports.

[25] Manca V (2017). The principles of informational genomics. Theor Comput Sci.

[26] Girvan M, Newman ME (2002). Community structure in social and biological networks. Proc Natl Acad Sci.

[27] Manber U, Myers G (1993). Suffix arrays: a new method for on-line string searches. SIAM J Comput.

[28] Abouelhoda MI, Kurtz S, Ohlebusch E (2002). The enhanced suffix array and its applications to genome analysis. International Workshop on Algorithms in Bioinformatics.

[29] Kurtz S, Narechania A, Stein JC, Ware D (2008). A new method to compute k-mer frequencies and its application to annotate large repetitive plant genomes. BMC Genomics.

[30] Bonnici V, Manca V (2015). Infogenomics tools: A computational suite for informational analysis of genomes. J Bioinforma Proteomics Rev.

[31] Rieck K, Laskov P (2008). Linear-time computation of similarity measures for sequential data. J Mach Learn Res.

[32] Qi J, Wang B, Hao B-I (2004). Whole proteome prokaryote phylogeny without sequence alignment: a k-string composition approach. J Mol Evol.

[33] Qi J, Luo H, Hao B (2004). Cvtree: a phylogenetic tree reconstruction tool based on whole genomes. Nucleic Acids Res.

[34] Stoye J, Evers D, Meyer F (1998). Rose: generating sequence families. Bioinformatics (Oxford, England).

[35] Baumdicker F, Hess WR, Pfaffelhuber P (2012). The infinitely many genes model for the distributed genome of bacteria. Genome Biol Evol.

[36] Brown T, Didelot X, Wilson DJ, De Maio N. Simbac: simulation of whole bacterial genomes with homologous recombination. Microbial Genomics. 2016; 2(1). 10.1099/mgen.0.000044 PMC5049688 27713837

