# Supplementary material for: PanDelos: a dictionary-based method for pan-genome content discovery
Source: BMC Bioinformatics. 2018 Nov 30;19(Suppl 15):437. doi: 10.1186/s12859-018-2417-6 (PMC6266927; doi:10.1186/s12859-018-2417-6)
Supplement: Supplementary file 1 — Supplementary materials of PanDelos: a dictionary-based method for pan-genome content discovery. (PDF 5543 kb) [file 12859_2018_2417_MOESM1_ESM.pdf]

Supplementary materials of PanDelos: a dictionary-based method for pan-genome content discovery

Tables 1, 2, 3 and 4 report a descriptive accession, together with the NCBI identifiers, of the isolates used to compare PanDelos to the state of the art approaches, Roary and EDGAR, for the discovery of pan-genome content. Annotations files, reporting peptide sequences and genomic coordinates of coding sequences (CDS), were downloaded from NCBI as GBK (GenBank data format) files and converted to GFF (General Feature Format) version 3 in order to be used as input for Roary. All the selected sequences are available on the EDGAR web interface. The selection includes 7 *Typhi* serotypes of the *Salmonella enterica* species (Table 1), 10 isolates of the *Escherichia coli* species (Table 2), 14 isolates of the *Xanthomonas* genus (Table 3) and 64 isolates belonging to the *Mycoplasma* genus (Table 4).

The composition of the four collections is reported in Figures 1, 2, 3 and 4. For the first three collections, the number of genetic sequences within each isolate is reported together with the total genome length, obtained as the sum of the lengths of the genes that the genome contains. The description also reports a distribution which informs for each gene length the number of genes having that specific length. In the description of the fourth dataset, the *Mycoplasma* isolates, genomic lengths and number of genes per genome are reported as a distribution, rather than giving the raw numbers. Phylogenetic distances and estimated phylogenetic trees of the four datasets are reported in Figures 5, 6, 7 and 8.

Figure 9 reports trend lines obtained by discovering pan-genome contents in the four real datasets. Table 5 shows the description of the genes detected as core by the three compared methodologies.

The dataset compositional distributions for the complete 2,000 individuals datasets generated by taking the *Mycoplasma genitaluium* G37 strain (NC_000908) isolate and using a locus variation probability equal to 0.5% is reported in Figure 11. Figure 12 shows the compositional distributions of the the 50 *roots* and 50 *leaves* extracted from the G37's population. The description is equipped with the traced pan-genomic distribution, that reports how many gene families are shared among a given number of genomes. Singletons are reported as belonging to only one genome, while dispensable families found in at most 2 isolates are reported in the corresponding counter for 2 genomes, and so forth up to the counter of core genomes. A similar composition was obtained for the synthetic collections generated with a locus variation equal to 1% and for the two further collections generated by taking the *Mycoplasma pneumoniae* M129 (NC_000912) isolate as common ancestor. The selection of the two common ancestors was made randomly. Figure 10 shows phylogenetic circular trees for the four synthetic populations of 2,000 genomes. Statistical properties of the phylogenetic tree of the population generated from the *Mycoplasma genitalium* G37, with a locus variation percentage equal to 0.5%, are given in Table 6. Similar properties are observed the three other synthetic populations. Details

regarding phylogenetic relationships and phylogenetic distances of the extracted datasets, *roots* and *leaves*, are shown in Figures 13, 15, 14, 16, 17, 19, 18 and 20. The figures show a circular tree on the top side of the image that reports the phylogenetic relationships among the extracted genome. Each genome is identified by a numerical ID. On the bottom side of the image, a heatmap reports the phylogenetic distances among the extracted genomes. The top-left color legend shows the mapping of the phylogenetic distances to the corresponding colors, and it also reports a density histogram that informs about the number of genome pairs within a specific distance interval. An agglomerative hierarchical clustering is computed over the reported distances by means of the complete-linkage method, and it is reported as a dendrogram on the top and the left side of the heatmap. The numerical identifiers shown on the right side of the heatmap correspond to the genome identifiers of the circular phylogenetic tree. Bench length on the circular tree does not represent phylogenetic distance. On the contrary, branch length if the dendrogram reports the relative hierarchical distance. Figure 21 shows the execution times of PanDelos and Roary over the four dataset extracted from the synthetic populations generated from the *Mycoplasma pneumoniae M129 genome.*

Phylogenetic distances have been computed by means of the CVTree software [1] *and a parallel implementation of it* [2]*. Hierarchical clustering and heatmap have been computed in R by means of the heatmap.2 function* [3] *located in the gplots package.*

Table 1: Identifiers of the 7 *Typhi* serotypes of the *Salmonella enterica* species.

	NCBI ID
Salmonella_enterica_subsp_enterica_serovar_Typhi_str_CT18	NC_003198
Salmonella_enterica_subsp_enterica_serovar_Typhi_str_P_stx_12	NC_016832
Salmonella_enterica_subsp_enterica_serovar_Typhi_strain_BL60006	NZ_LT882486
Salmonella_enterica_subsp_enterica_serovar_Typhi_str_Ty21a	NC_021176
Salmonella_enterica_subsp_enterica_serovar_Typhi_str_Ty2	NC_004631
Salmonella_enterica_subsp_enterica_serovar_Typhi_strain_B_SF_13_03_195	NZ_CP012151
Salmonella_enterica_subsp_enterica_serovar_Typhi_strain_PM016_13	NZ_CP012091

Table 2: Identifiers of the 10 *Escherichia coli* isolates.

	NCBI ID
Escherichia_coli_str_K-12_substr_MG1655	NC_000913
Escherichia_coli_O157:H7_str_EC4115	NC_011353
Escherichia_coli_O157:H7_str_EDL933	NC_002655
Escherichia_coli_O157:H7_str_Sakai	NC_002695
Escherichia_coli_CFT073	NC_004431
Escherichia_coli_UTI89	NC_007946
Escherichia_coli_536	NC_008253
Escherichia_coli_APEC_O1	NC_008563
Escherichia_coli_HS	NC_009800
Escherichia_coli_E24377A	NC_009801

[1] *https://github.com/ghzuo/CVTree*
[2] *https://github.com/jacobjmarks/cvtree-parallel*
[3] *https://www.rdocumentation.org/packages/gplots/versions/3.0.1/topics/heatmap.2*

Table 3: Identifiers of the 14 *Xanthomonas* isolates.

	NCBI ID
Xanthomonas_axonopodis_Xac29_1	NC_020800
Xanthomonas_axonopodis_pv_citri_str_306	NC_003919
Xanthomonas_campestris_pv_vesicatoria_str_85_10	NC_007508
Xanthomonas_citri_subsp_citri_Aw12879	NC_020815
Xanthomonas_albilineans_GPE_PC73	NC_013722
Xanthomonas_axonopodis_pv_citrumelo_F1	NC_016010
Xanthomonas_campestris_pv_campestris_str_8004	NC_007086
Xanthomonas_campestris_pv_campestris_str_ATCC_33913	NC_003902
Xanthomonas_campestris_pv_campestris_strain_B100	NC_010688
Xanthomonas_campestris_pv_raphani_756C	NC_017271
Xanthomonas_oryzae_pv_oryzae_KACC_10331	NC_006834
Xanthomonas_oryzae_pv_oryzae_MAFF_311018	NC_007705
Xanthomonas_oryzae_pv_oryzae_PXO99A	NC_010717
Xanthomonas_oryzae_pv_oryzicola_BLS256	NC_017267

Total gen length	Sequences per genome
1,252,204	3,674
1,321,296	4,031
1,047,863	2,966
1,447,776	4,297
1,422,278	4,236
1,373,700	4,055
1,392,527	4,237
1,447,868	4,321
1,431,604	4,179
1,475,102	4,447
1,397,466	4,066
1,254,716	3,940
1,242,874	4,055
1,421,436	4,255

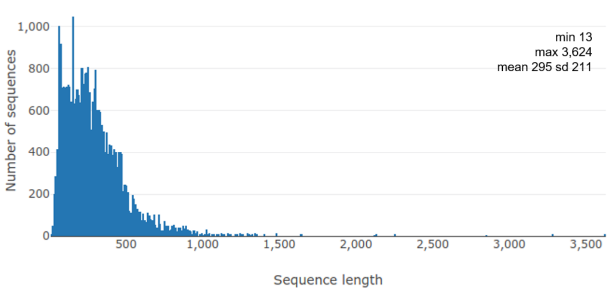

Figure 1: Composition of the *Salmonella enterica Typhi* dataset. The figure reports the genome lengths, obtained by summing the lengths of the genes in each genome, and the count of gene sequences for the 7 analyzed genomes. The chart of the right side reports the distribution of gene sequence lengths for the entire dataset.

Total gene length	Sequences per genome
1,592,414	5,550
1,571,498	5,428
1,481,733	4,989
1,418,100	4,623
1,486,521	4,895
1,438,945	4,796
1,339,982	4,407
1,569,952	5,200
1,474,499	4,952
1,312,010	4,140

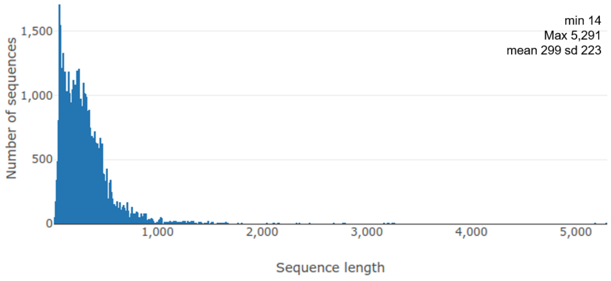

Figure 2: Composition of the *Escherichia coli* dataset. The figure reports the genome lengths, obtained by summing the lengths of the genes in each genome, and the count of gene sequences for the 10 analyzed genomes. The chart of the right side reports the distribution of gene sequence lengths for the entire dataset.

Total gene length	Sequences per genome
1,252,204	3,674
1,321,296	4,031
1,047,863	2,966
1,447,776	4,297
1,422,278	4,236
1,373,700	4,055
1,392,527	4,237
1,447,868	4,321
1,431,604	4,179
1,475,102	4,447
1,397,466	4,066
1,254,716	3,940
1,242,874	4,055
1,421,436	4,255

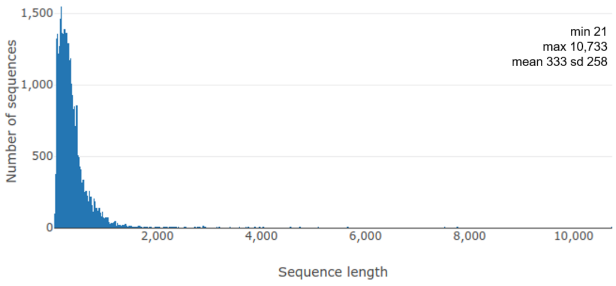

Figure 3: Composition of the *Xanthomonas campestris* dataset. The figure reports the genome lengths, obtained by summing the lengths of the genes in each genome, and the count of gene sequences for the 14 analyzed genomes. The chart of the right side reports the distribution of gene sequence lengths for the entire dataset.

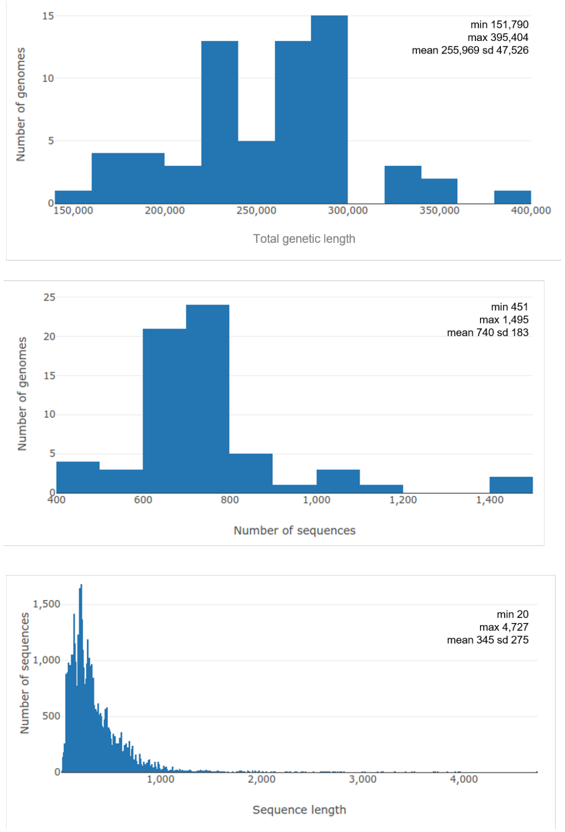

Figure 4: Composition of the *Mycoplasma* dataset.

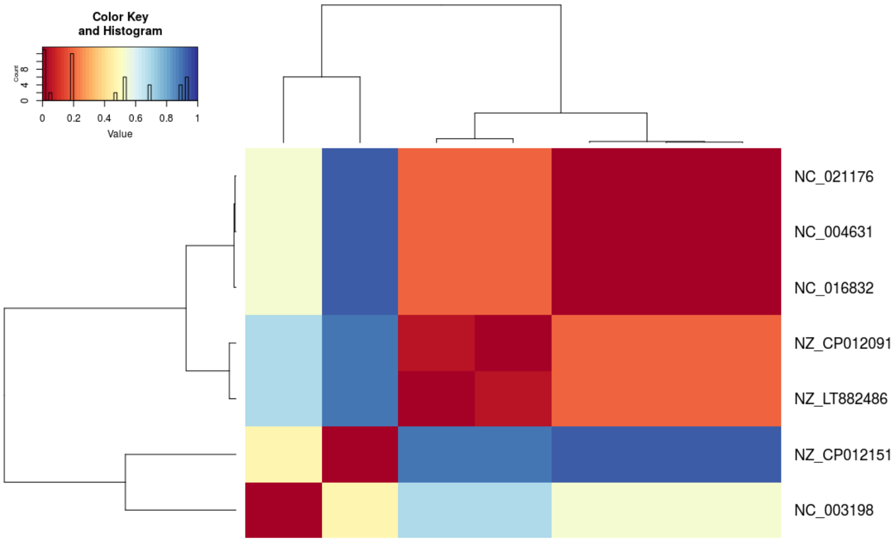

Figure 5: Heatmap reporting the phylogenetic distances among the 7 analyzed *Salmonella enterica* genomes. The top-left color legend shows the mapping of the phylogenetic distances to the corresponding colors, and it also reports a density histogram that informs about the number of genome pairs within a specific distance range. An agglomerative hierarchical clustering is computed by means of the complete-linkage method over the reported distances and reported as a dendrogram on the top and left side of the heatmap.

Table 4: Identifiers of the 64 *Mycoplasma* isolates.

	NCBI ID
Mycoplasma_agalactiae_PG2	NC_009497
Mycoplasma_agalactiae_strain_5632	NC_013948
Mycoplasma_arthritidis_158L3_1	NC_011025
Mycoplasma_bovis_HB0801	NC_018077
Mycoplasma_bovis_Hubei_1	NC_015725
Mycoplasma_bovis_PG45	NC_014760
Mycoplasma_capricolum_subsp_capricolum_ATCC_27343	NC_007633
Mycoplasma_conjunctivae_HRC_581	NC_012806
Mycoplasma_crocodyli_MP145	NC_014014
Mycoplasma_cynos_C142	NC_019949
Mycoplasma_fermentans_JER	NC_014552
Mycoplasma_fermentans_M64	NC_014921
Mycoplasma_fermentans_PG18	NC_021002
Mycoplasma_gallisepticum_CA06_2006052_5_2P	NC_018412
Mycoplasma_gallisepticum_NC06_2006080_5_2P	NC_018411
Mycoplasma_gallisepticum_NC08_2008031_4_3P	NC_018413
Mycoplasma_gallisepticum_NC95_13295_2_2P	NC_018407
Mycoplasma_gallisepticum_NC96_1596_4_2P	NC_018408
Mycoplasma_gallisepticum_NY01_2001047_5_1P	NC_018409
Mycoplasma_gallisepticum_S6	NC_023030
Mycoplasma_gallisepticum_VA94_7994_1_7P	NC_018406
Mycoplasma_gallisepticum_WI01_2001043_13_2P	NC_018410
Mycoplasma_gallisepticum_str_F	NC_017503
Mycoplasma_gallisepticum_str_Rhigh	NC_017502
Mycoplasma_gallisepticum_str_Rlow	NC_004829
Mycoplasma_genitalium_G37_strain_G_37	NC_000908
Mycoplasma_genitalium_M2288	NC_018498
Mycoplasma_genitalium_M2321	NC_018495
Mycoplasma_genitalium_M6282	NC_018496
Mycoplasma_genitalium_M6320	NC_018497
Mycoplasma_haemocanis_str_Illinois	NC_016638
Mycoplasma_haemofelis_Ohio2	NC_017520
Mycoplasma_haemofelis_str_Langford_1	NC_014970
Mycoplasma_hominis_ATCC_23114_strain_PG21	NC_013511
Mycoplasma_hyopneumoniae_168_L	NC_021283
Mycoplasma_hyopneumoniae_168	NC_017509
Mycoplasma_hyopneumoniae_232	NC_006360
Mycoplasma_hyopneumoniae_7422	NC_021831
Mycoplasma_hyopneumoniae_7448	NC_007332
Mycoplasma_hyopneumoniae_J	NC_007295
Mycoplasma_hyorhinis_DBS_1050	NC_022807
Mycoplasma_hyorhinis_GDL_1	NC_016829
Mycoplasma_hyorhinis_HUB_1	NC_014448
Mycoplasma_hyorhinis_MCLD	NC_017519
Mycoplasma_hyorhinis_SK76	NC_019552
Mycoplasma_leachii_99_014_6	NC_017521
Mycoplasma_leachii_PG50	NC_014751
Mycoplasma_mobile_163K	NC_006908
Mycoplasma_mycoides_subsp_mycoides_SC_str_Gladysdale	NC_021025
Mycoplasma_mycoides_subsp_mycoides_SC_str_PG1	NC_005364
Mycoplasma_ovis_str_Michigan	NC_023062
Mycoplasma_parvum_str_Indiana	NC_022575
Mycoplasma_penetrans_HF_2	NC_004432
Mycoplasma_pneumoniae_309	NC_016807
Mycoplasma_pneumoniae_FH	NC_017504
Mycoplasma_pneumoniae_M129_B7	NC_020076
Mycoplasma_pneumoniae_M129	NC_000912
Mycoplasma_pulmonis_UAB_CTIP	NC_002771
Mycoplasma_putrefaciens_KS1	NC_015946
Mycoplasma_putrefaciens_Mput9231	NC_021083
Mycoplasma_suis_KI3806_strain_KI_3806	NC_015153
Mycoplasma_suis_str_Illinois	NC_015155
Mycoplasma_synoviae_53	NC_007294
Mycoplasma_wenyonii_str_Massachusetts	NC_018149

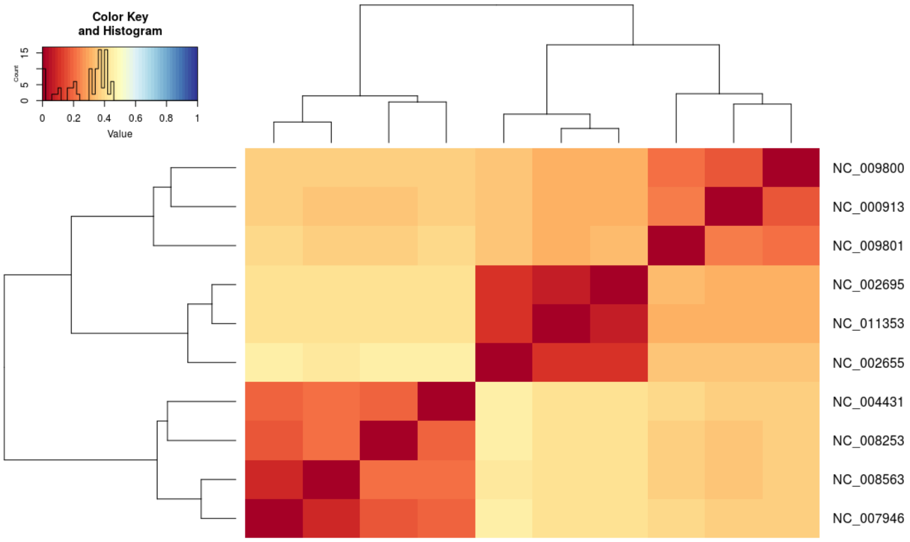

Figure 6: Heatmap reporting the phylogenetic distances among the 10 analyzed *Escheirchia coli* genomes. The top-left color legend shows the mapping of the phylogenetic distances to the corresponding colors, and it also reports a density histogram that informs about the number of genome pairs within a specific distance range. An agglomerative hierarchical clustering is computed by means of the complete-linkage method over the reported distances and reported as a dendrogram on the top and left side of the heatmap.

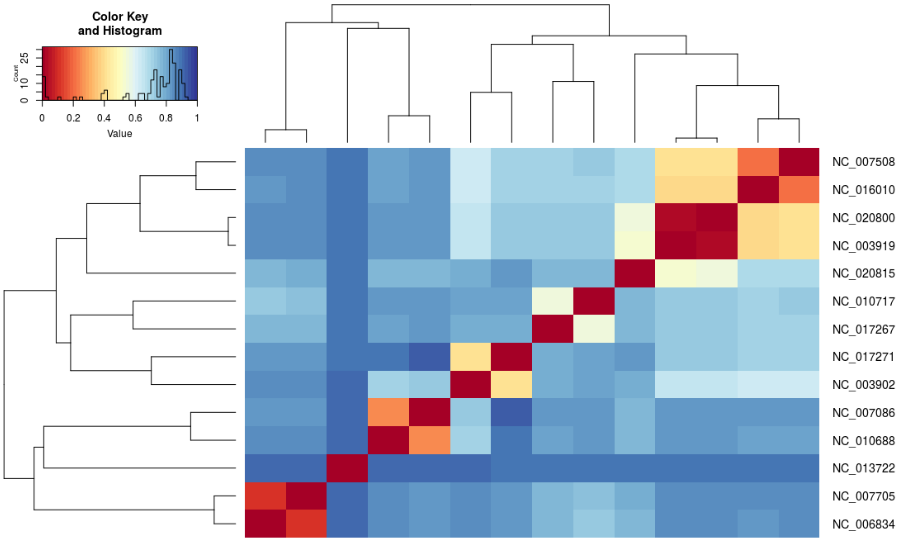

Figure 7: Heatmap reporting the phylogenetic distances among the 14 analyzed *Xanthomonas campestris* genomes. The top-left color legend shows the mapping of the phylogenetic distances to the corresponding colors, and it also reports a density histogram that informs about the number of genome pairs within a specific distance range. An agglomerative hierarchical clustering is computed by means of the complete-linkage method over the reported distances and reported as a dendrogram on the top and left side of the heatmap.

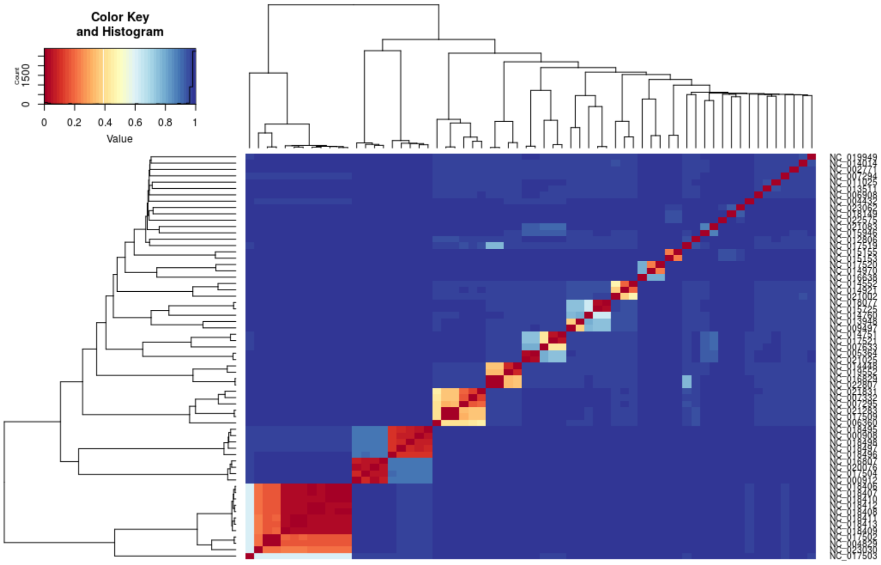

Figure 8: Heatmap reporting the phylogenetic distances among the 64 analyzed *Mycoplasma* genomes. The top-left color legend shows the mapping of the phylogenetic distances to the corresponding colors, and it also reports a density histogram that informs about the number of genome pairs within a specific distance range. An agglomerative hierarchical clustering is computed by means of the complete-linkage method over the reported distances.

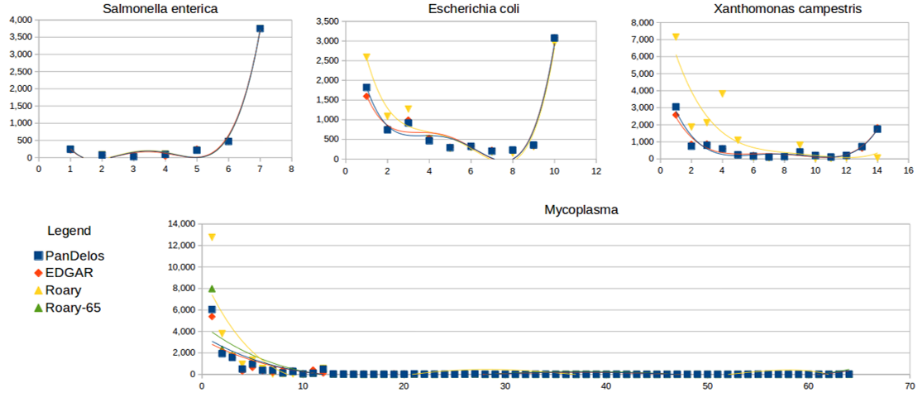

Figure 9: Scatter-plots of pan-genome trends reporting number of gene families that have been found in a specific number of input genome. Trends are reported for each of the four real benchmarks. Trend lines are calculated by fitting a fourth degree polynomial function from the data.

Table 5: List of core genes fond by the three approaches in the *Mycoplasma* dataset. Core gene shared by PanDelos and EDGAR are marked with the symbol '*'.

PanDelos
30S ribosomal protein S13
30S ribosomal protein S19
30S ribosomal protein S7 *
50S ribosomal protein L11 *
50S ribosomal protein L13
50S ribosomal protein L14 *
50S ribosomal protein L2 *
50S ribosomal protein L20 *
50S ribosomal protein L27 *
50S ribosomal protein L35
aldehyde dehydrogenase
ATP-dependent zinc metalloprotease FtsH
cobalt transporter ATP-binding protein
DNA topoisomerase IV subunit B
DNA-directed RNA polymerase subunit beta
elongation factor Tu *
ATP synthase subunit alpha *
ATP synthase subunit beta *
multiple annotations
phosphocarrier protein HPr
phosphoglycerate kinase
type I glyceraldehyde-3-phosphate dehydrogenase *
EDGAR
30S ribosomal protein S7 *
30S ribosomal protein S11
30S ribosomal protein S19
30S ribosomal protein S4
50S ribosomal protein L11 *
50S ribosomal protein L14 *
50S ribosomal protein L2 *
50S ribosomal protein L20 *
50S ribosomal protein L27 *
50S ribosomal protein L28
elongation factor Tu *
ATP synthase subunit alpha *
ATP synthase subunit beta *
type I glyceraldehyde-3-phosphate dehydrogenase *
Top 20 Roary (with 65% BLAST identity)
50S ribosomal protein L14 (64)
50S ribosomal protein L34 (64)
F0F1 ATP synthase subunit beta (63)
30S ribosomal protein S13 (63)
50S ribosomal protein L27 (61)
30S ribosomal protein S9 (59)
50S ribosomal protein L35 (56)
30S ribosomal protein S12 (56)
hypothetical protein (56)
50S ribosomal protein L22 (55)
50S ribosomal protein L33 (48)
50S ribosomal protein L11 (44)
elongation factor Tu (44)
translation initiation factor IF-1 (43)
ATP synthase subunit C (42)
preprotein translocase subunit SecG (42)
50S ribosomal protein L32 (37)
50S ribosomal protein L21 (34)
30S ribosomal protein S17 (34)
50S ribosomal protein L11 (33)

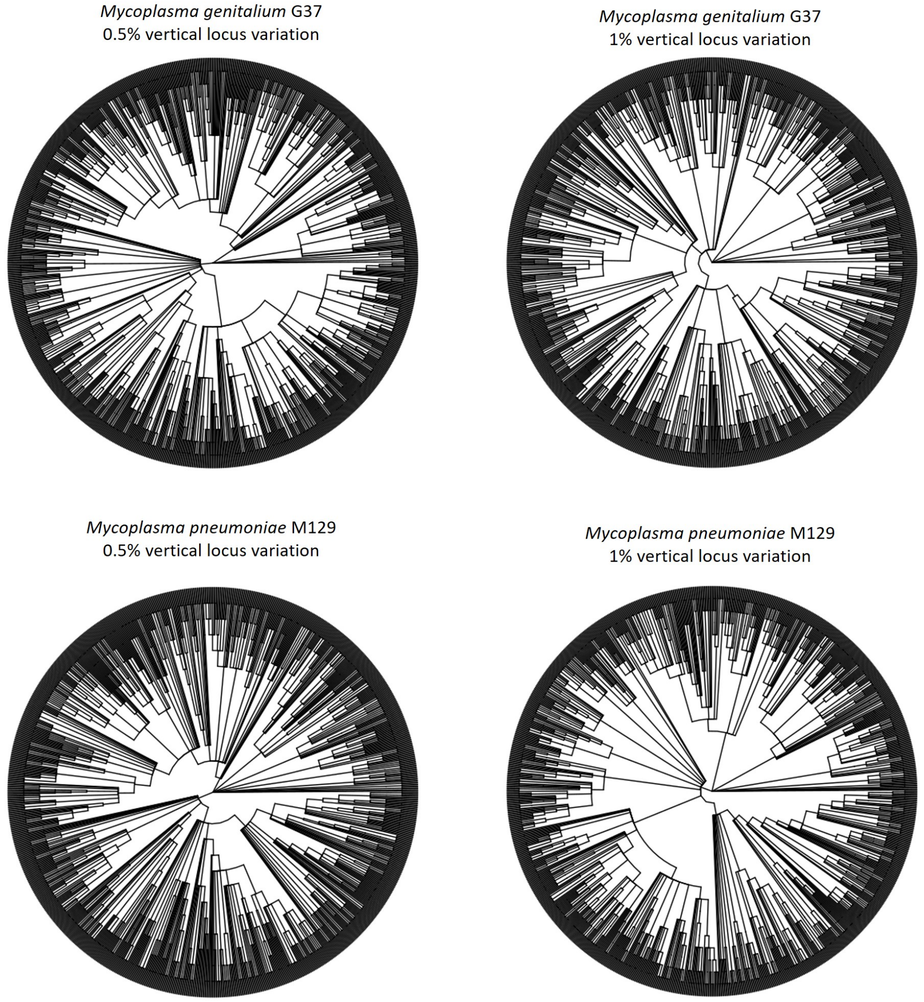

Figure 10: Phylogenetic trees of the four synthetic population obtained by synthetically evolving two real *Mycoplasma* reference genome, namely the *Mycoplasma genitalium* G37 and the *Mycoplasma pneumoniae* M129 genomes. Two populations have been evolved by using a locus percentage variation during vertical gene transmission equal to 0.5%, and two other populations have been evolved by using a percentage equal to 1%. All the synthetic leaf genomes are drawn at the margin of the circular tree. Branch length does not represent phylogenetic distance.

Table 6: Structural properties of the evolutionary tree of the synthetic population generated from the *Mycoplasma genitalium* G37 with a locus variation percentage equal to 0.5%. The population contains 2,000 genomes, 973 of them have at least one progeny and 1,027 are leaves. Similar trends are observed the three other synthetic populations.

Depth	Genomes	Mean degree (sd)	Leaves
0	1	7.00 (0.00)	0
1	7	5.33 (4.50)	1
2	32	3.60 (2.27)	7
3	90	2.63 (1.84)	36
4	142	2.47 (1.83)	54
5	217	2.30 (1.61)	109
6	248	2.01 (1.32)	111
7	275	1.97 (1.46)	134
8	278	2.00 (1.27)	147
9	262	1.77 (1.05)	147
10	203	1.49 (0.77)	124
11	118	1.51 (0.87)	69
12	74	1.33 (0.64)	50
13	32	1.56 (0.88)	23
14	14	1.20 (0.45)	9
15	6	1.00 (0.00)	5
16	1	0.00 (0.00)	1

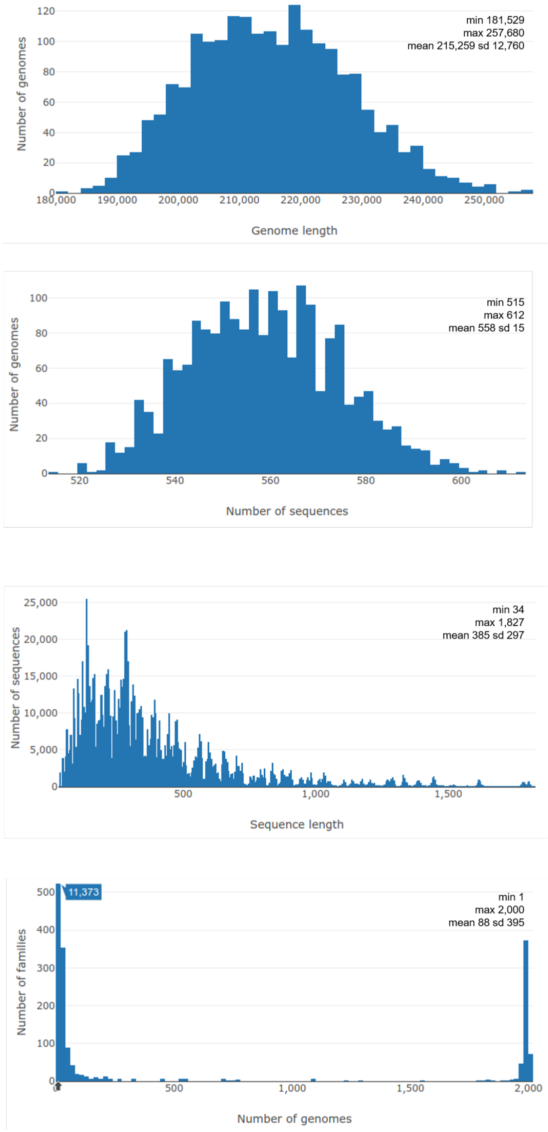

Figure 11: Composition of one of the two synthetic populations generated by starting from the real the *Mycoplasma genitalium* G37 genome. The dataset contains 2,000 genomes. The total genome lengths vary from 181 kilobases to 257 kilobases and their representation shapes a unimodal distribution. A similar shape is obtained by the distribution that informs how many gene sequences are contained in each genome, varying from 515 to 612. A different shape is obtained for the distribution of the genetic sequences lengths, that vary from 34 to 1,827 amino acids. There are 11 thousands singleton genes and 71 core genes. The rest of the gene families are present in more than 1 but less than 2,000 genomes. The trend is shown by the distribution on the bottom side of the figure. The distribution associates to any possible counting of genomes, from 1 to 2,000, the number of families that are present in exactly that specific number of individuals.

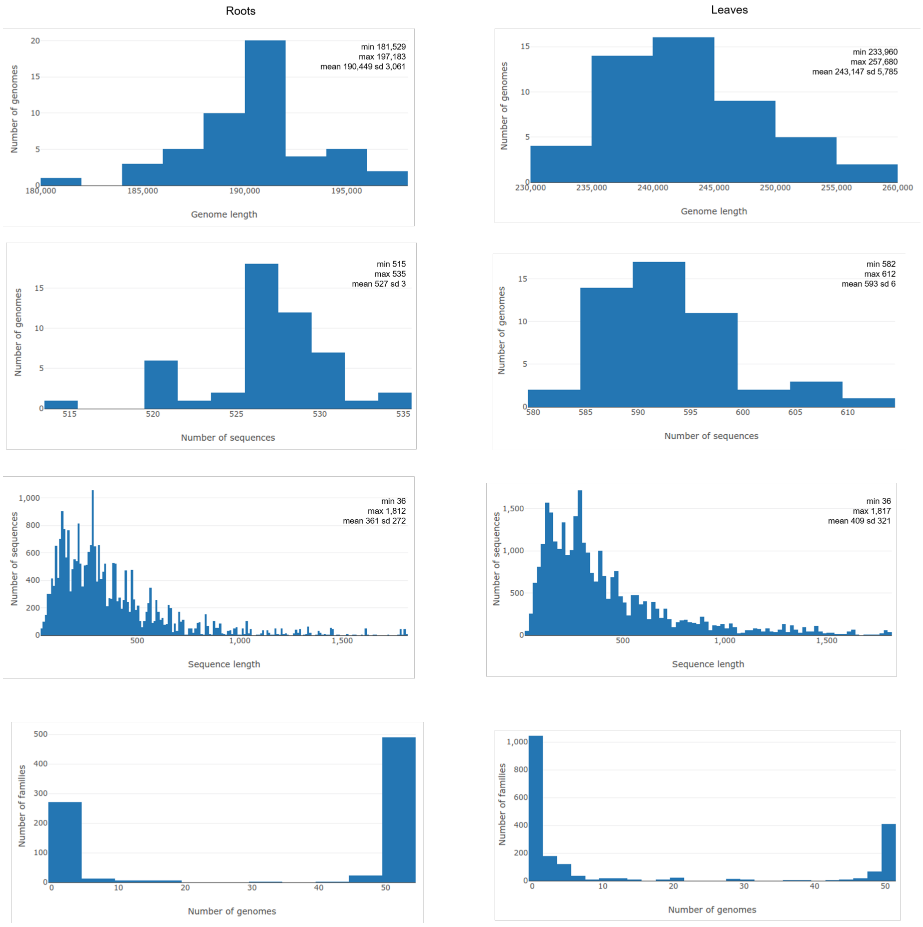

Figure 12: Composition of one of the 50 *roots* and 50 *leaves* datasets extracted from one of the synthetic populations generated from the *Mycoplasma genitalium* G37.

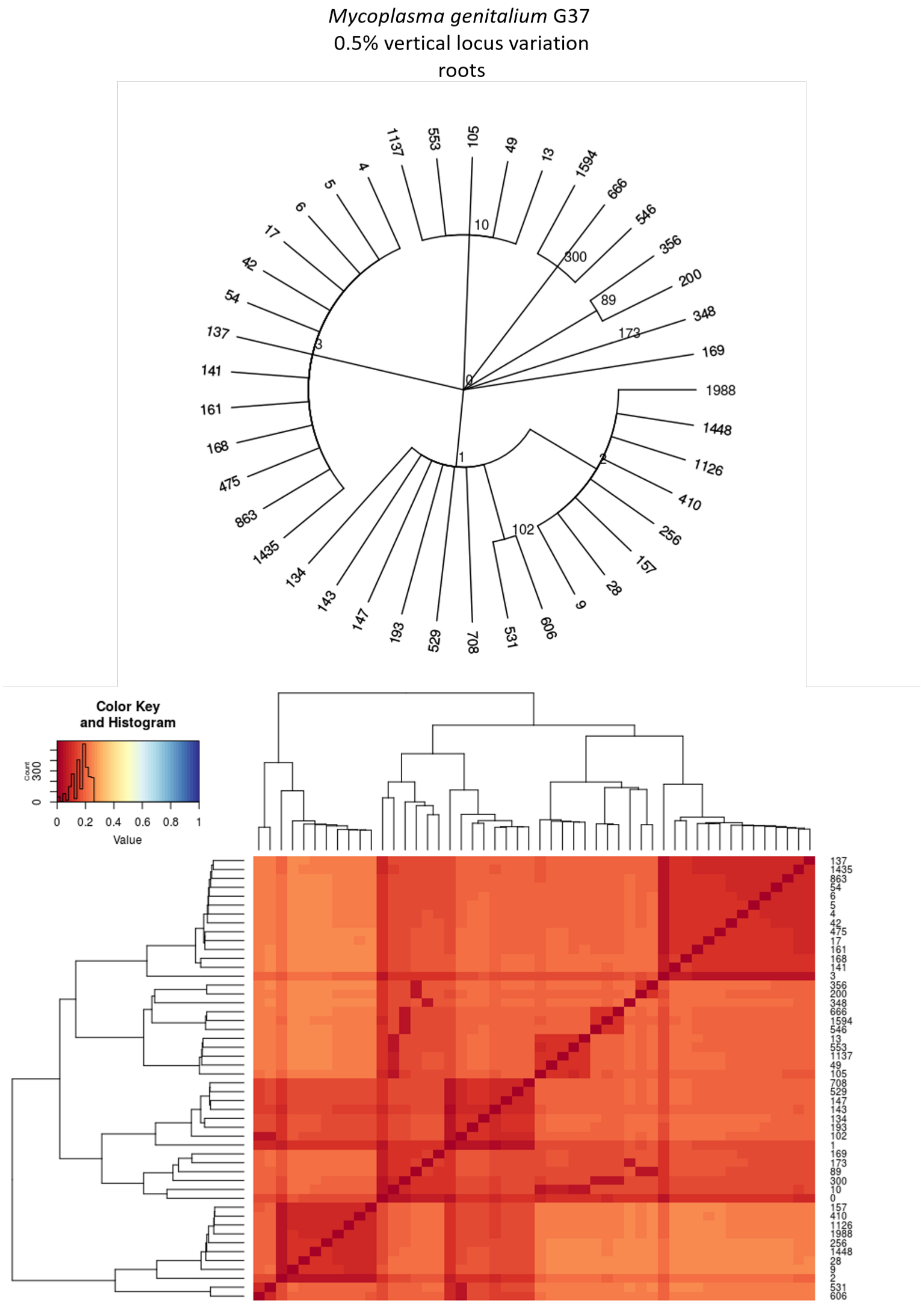

Figure 13: Phylogenetic trees of the dataset extracted from the synthetic population rooted on the real genome *Mycoplasma genitalum* G37 and evolved with a 0.5% locus variation during vertical gene transmission. The *roots* of the phylogenetic tree, namely the genomes more closed the root of the tree, are extracted.

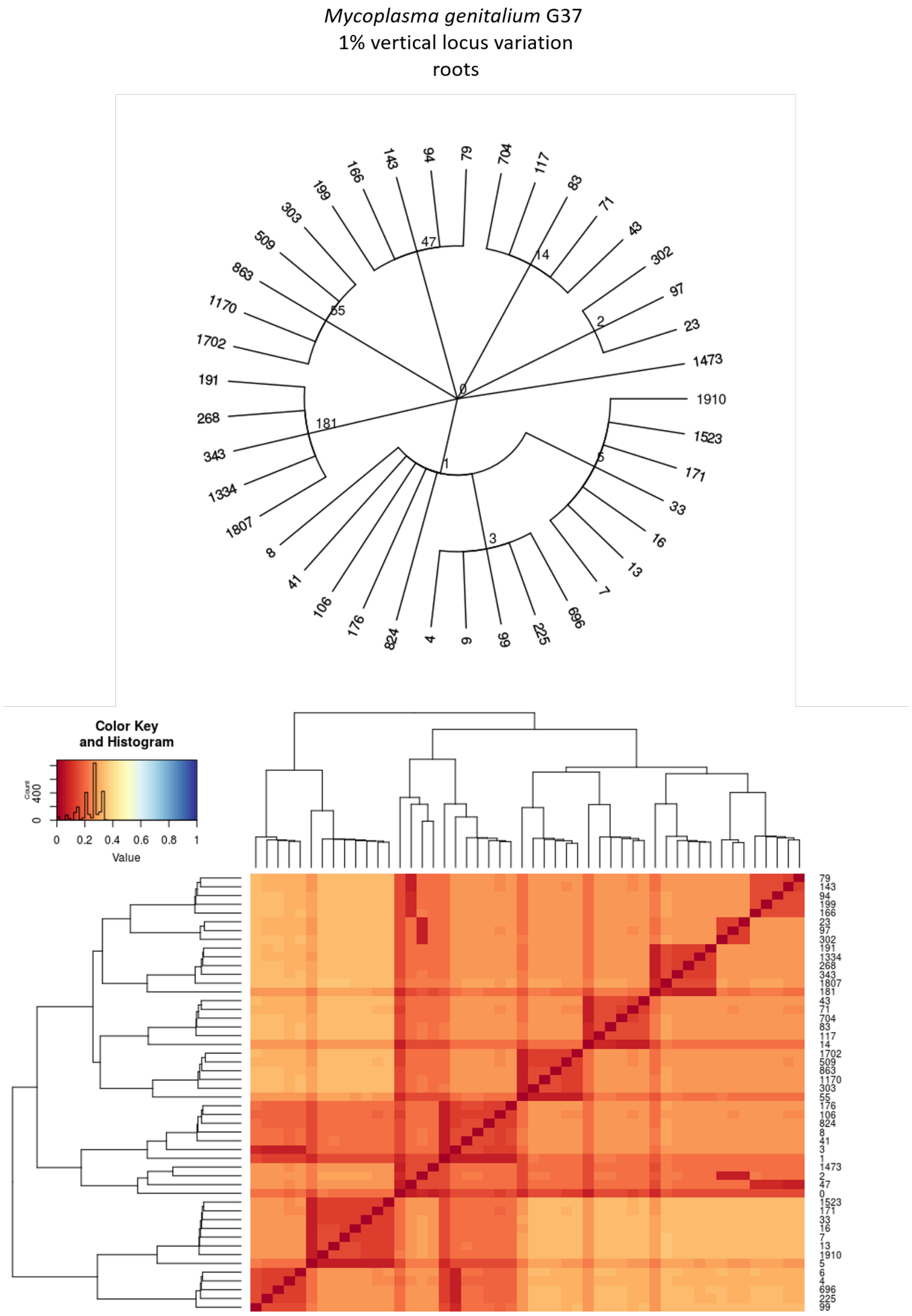

Figure 14: Phylogenetic trees of the dataset extracted from the synthetic population rooted on the real genome *Mycoplasma genitalum* G37 and evolved with a 1% locus variation during vertical gene transmission. The *roots* of the phylogenetic tree, namely the genomes more closed the root of the tree, are extracted.

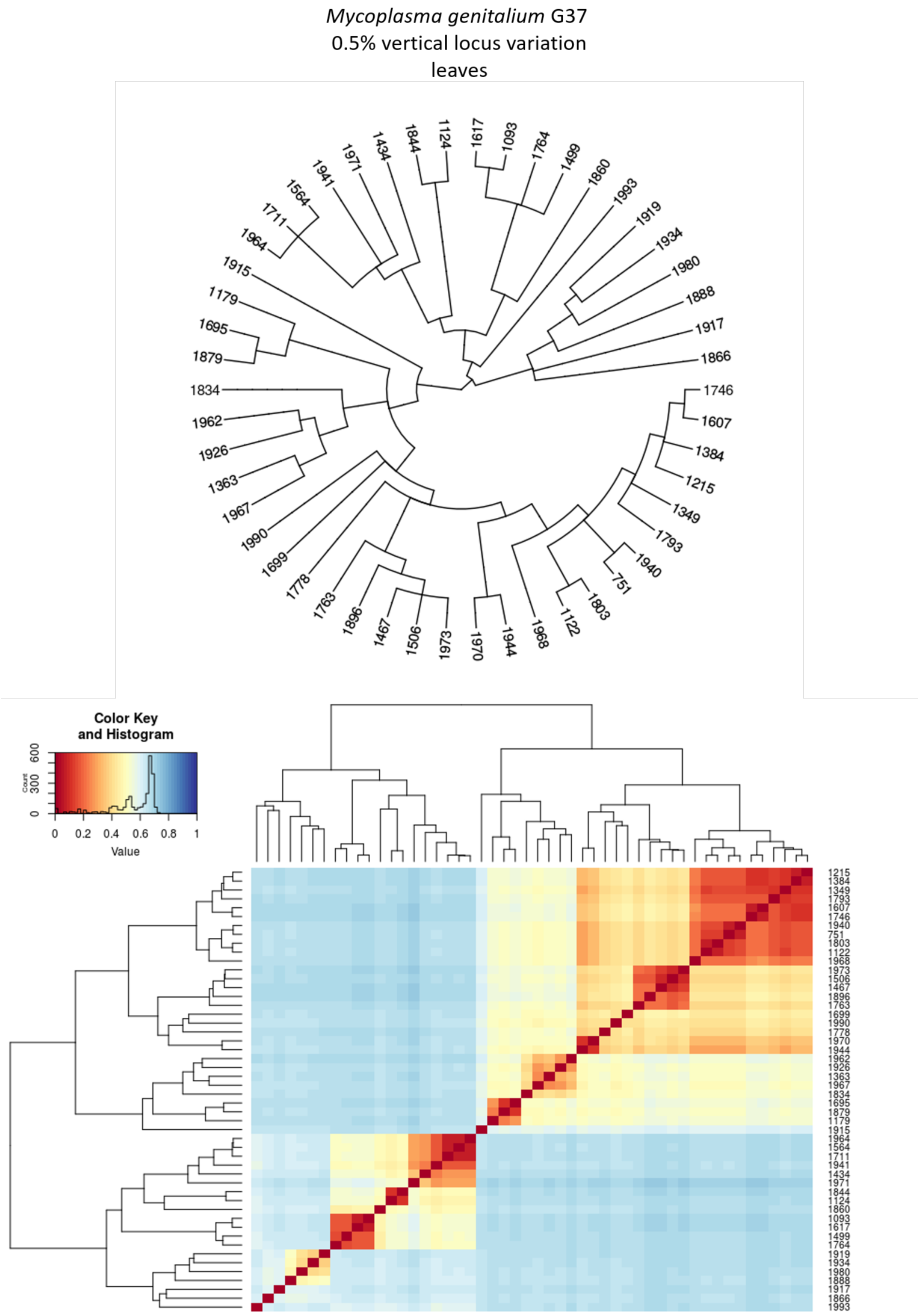

Figure 15: Phylogenetic trees of the dataset extracted from the synthetic population rooted on the real genome *Mycoplasma genitalum* G37 and evolved with a 0.5% locus variation during vertical gene transmission. The *leaves* of the phylogenetic tree, namely the genomes without a progeny, are extracted randomly.

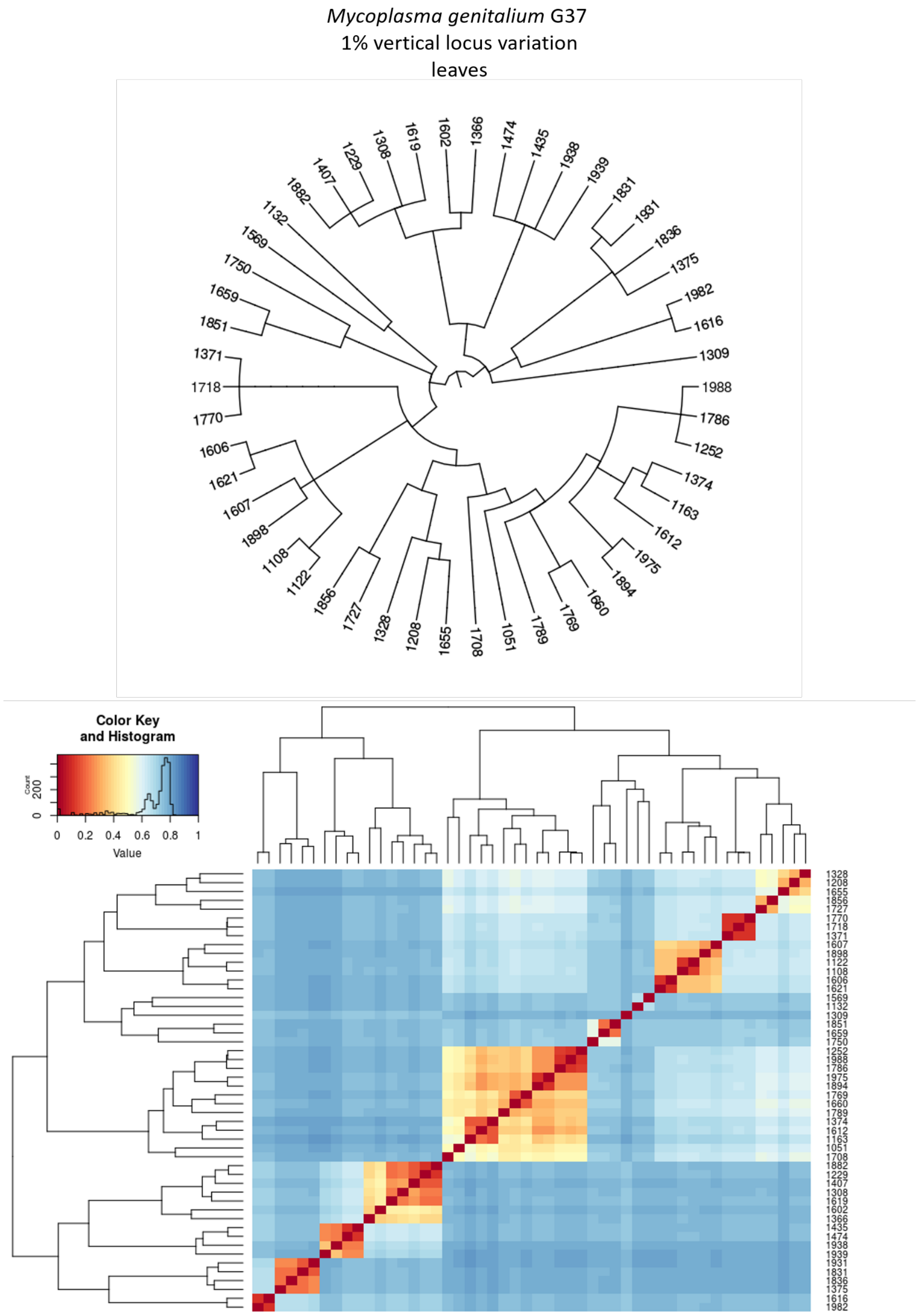

Figure 16: Phylogenetic trees of the dataset extracted from the synthetic population rooted on the real genome *Mycoplasma genitalum* G37 and evolved with a 1% locus variation during vertical gene transmission. The *leaves* of the phylogenetic tree, namely the genomes without a progeny, are extracted randomly.

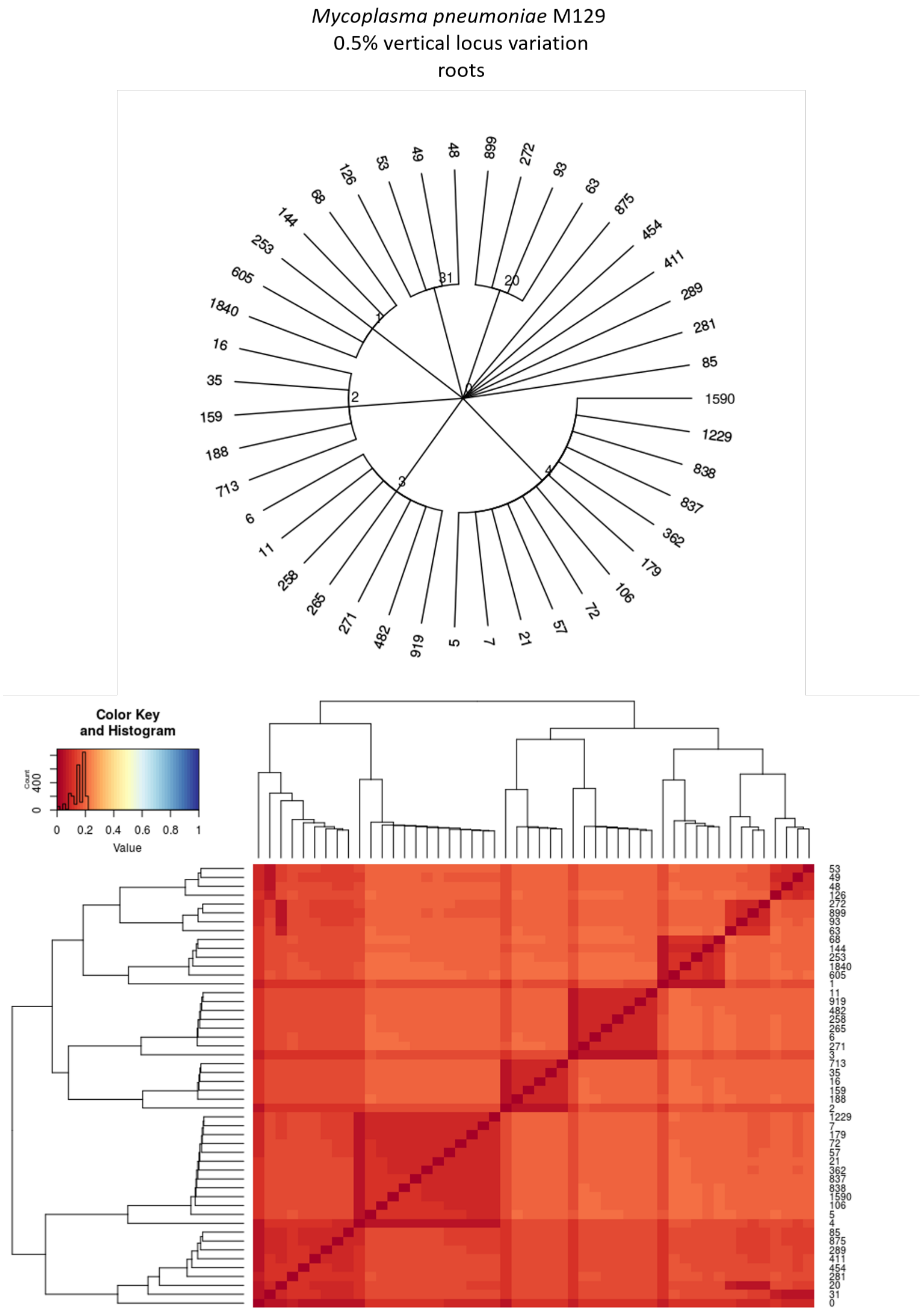

Figure 17: Phylogenetic trees of the dataset extracted from the synthetic population rooted on the real genome *Mycoplasma pneumoniae* M129 and evolved with a 0.5% locus variation during vertical gene transmission. The *roots* of the phylogenetic tree, namely the genomes more closed the root of the tree, are extracted

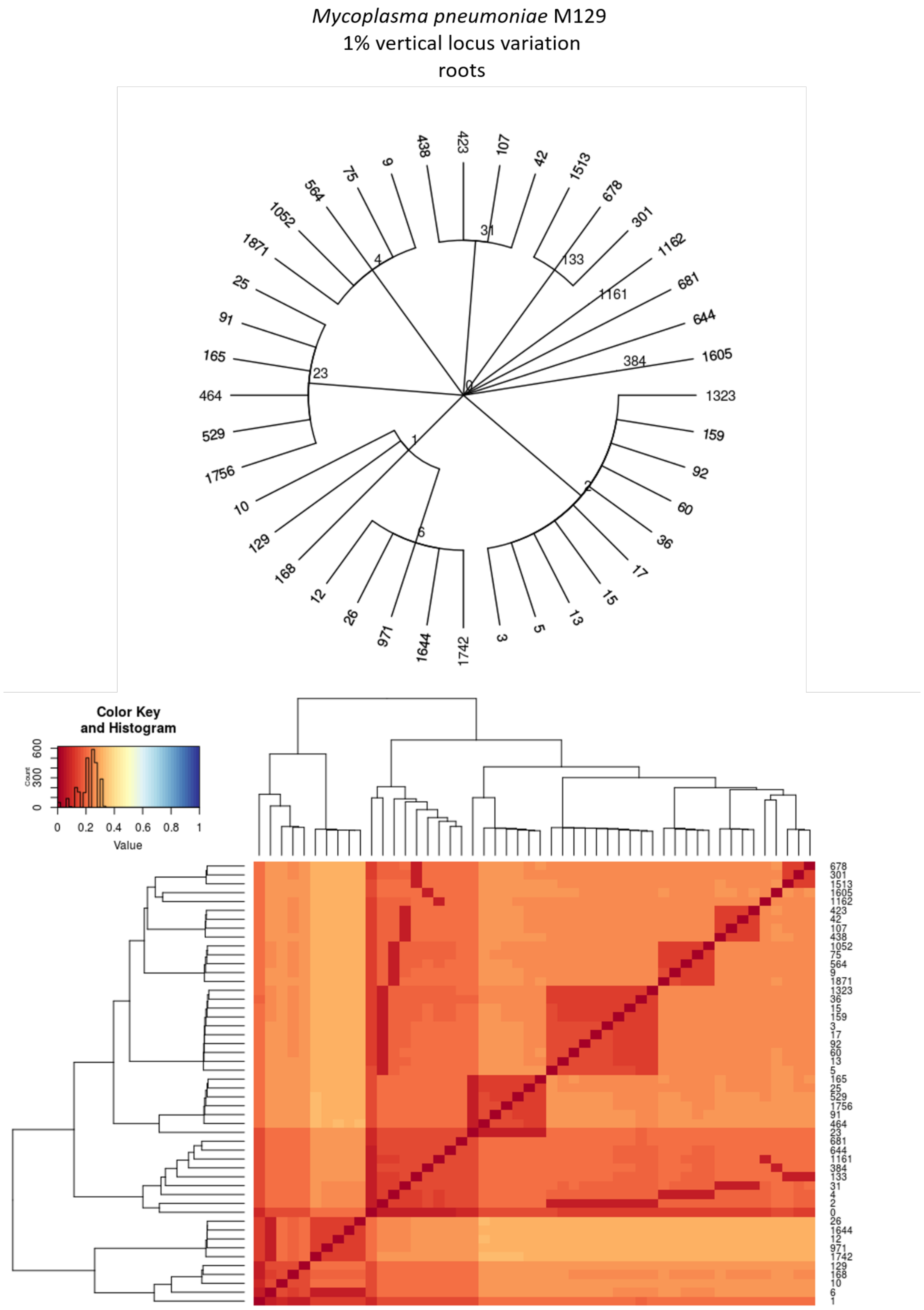

Figure 18: Phylogenetic trees of the dataset extracted from the synthetic population rooted on the real genome *Mycoplasma pneumoniae* M129 and evolved with a 1% locus variation during vertical gene transmission. The *roots* of the phylogenetic tree, namely the genomes more closed the root of the tree, are extracted.

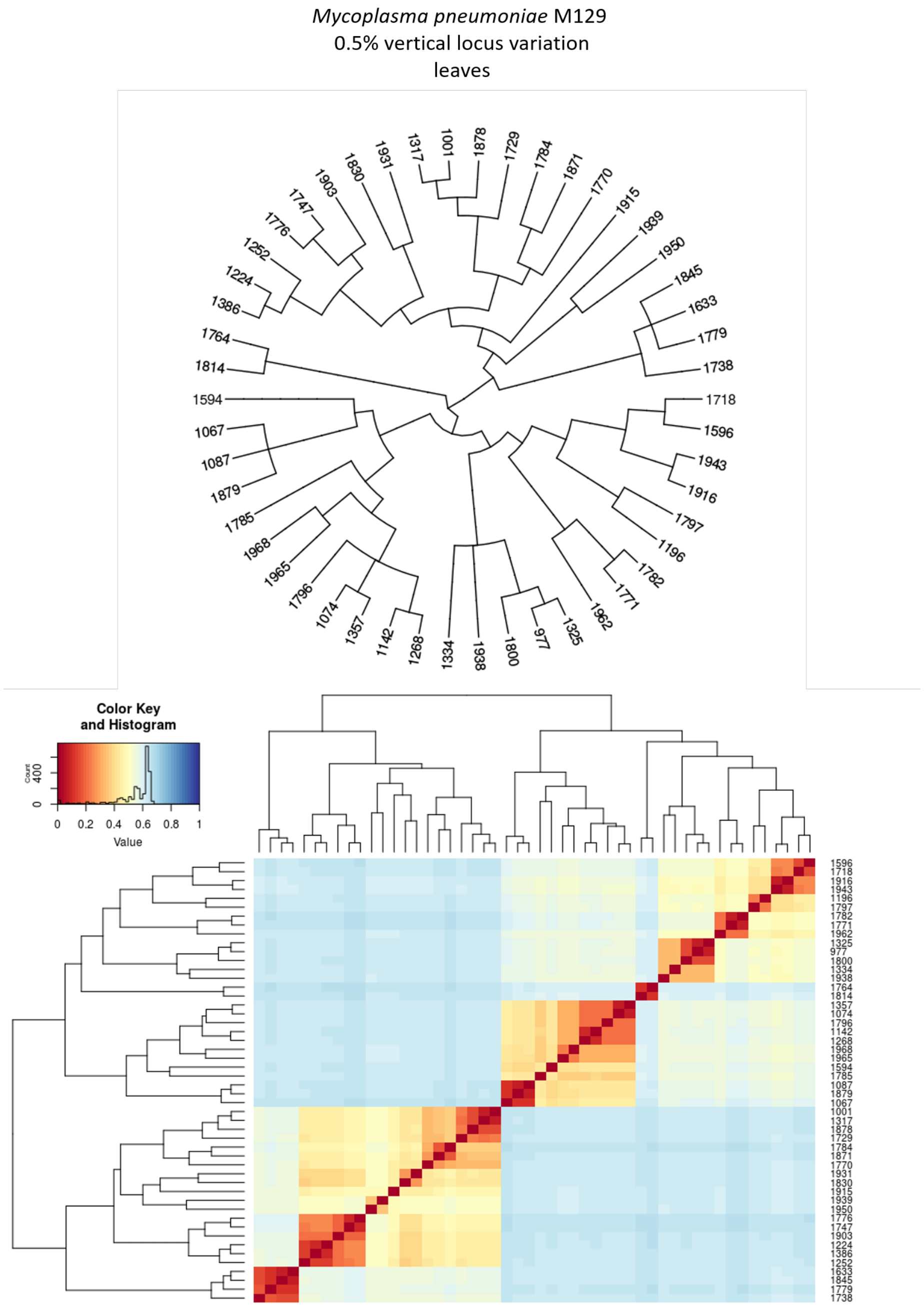

Figure 19: Phylogenetic trees of the dataset extracted from the synthetic population rooted on the real genome *Mycoplasma pneumoniae* M129 and evolved with a 0.5% locus variation during vertical gene transmission. The *leaves* of the phylogenetic tree, namely the genomes without a progeny, are extracted randomly.

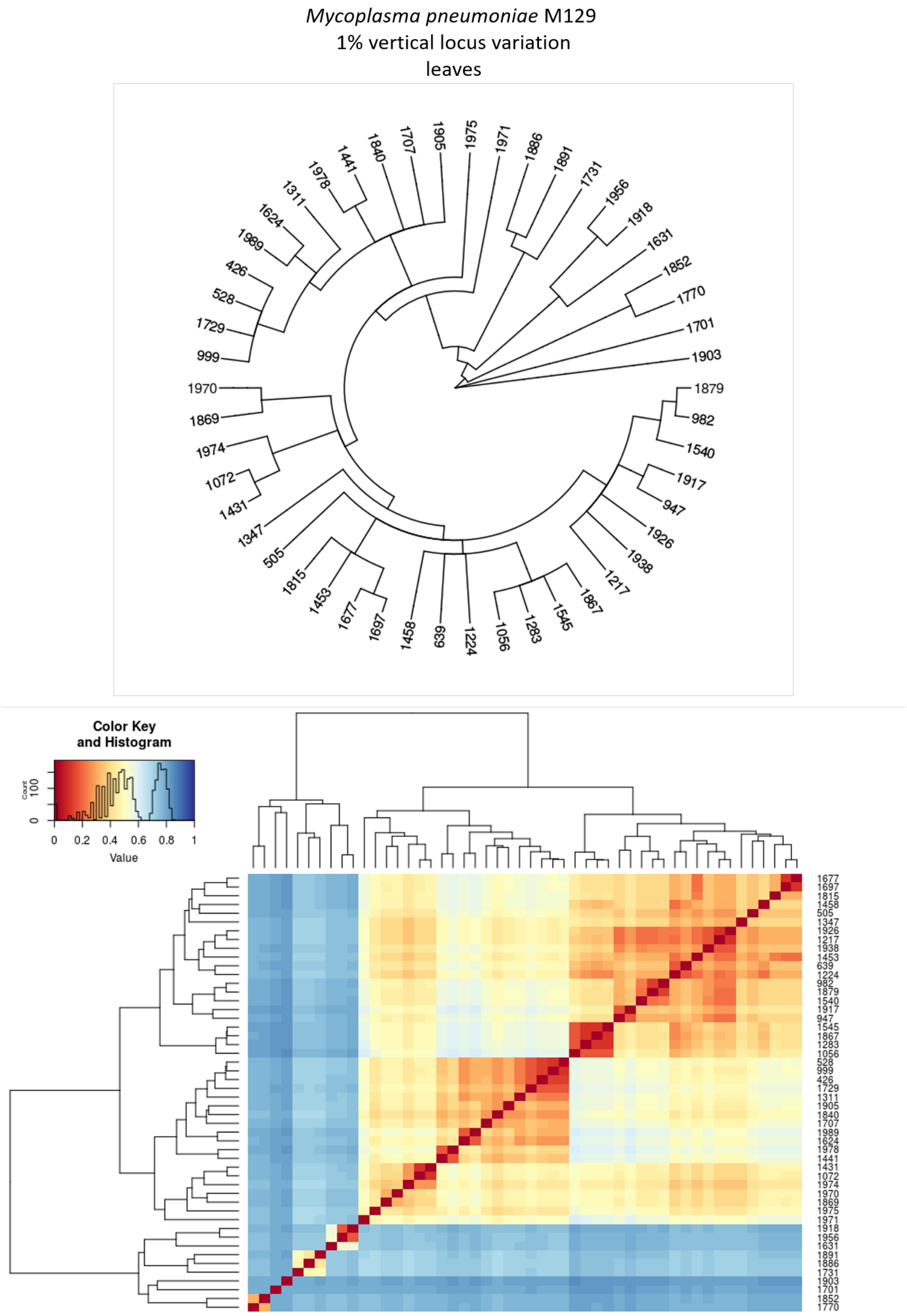

Figure 20: Phylogenetic trees of the dataset extracted from the synthetic population rooted on the real genome *Mycoplasma pneumoniae* M129 and evolved with a 1% locus variation during vertical gene transmission. The *leaves* of the phylogenetic tree, namely the genomes without a progeny, are extracted randomly.

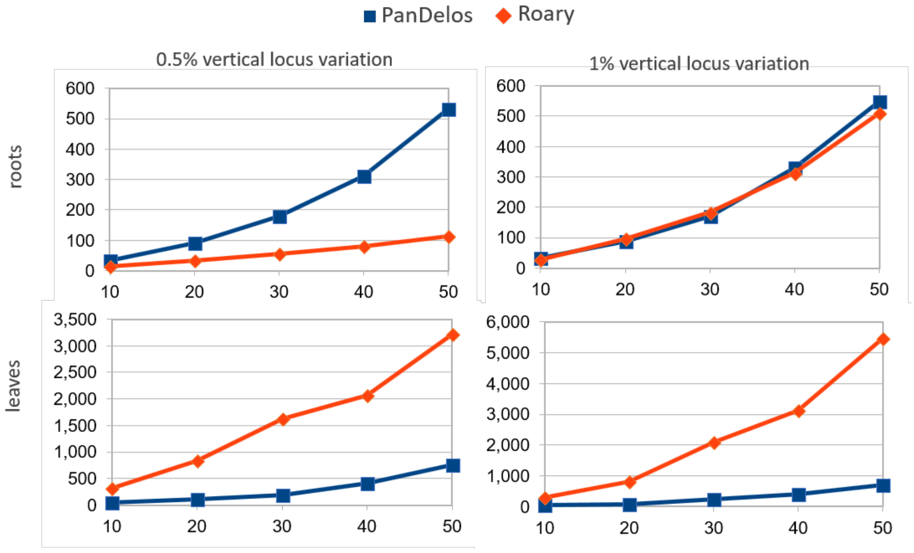

Figure 21: Execution times of PanDelos and Roary over the four synthetic datasets extracted form the ones generated from the *Mycoplasma pneumoniae* M129 genome. Time requirements have been measured by taking into account five different amounts of analyzed genomes, from 10 to 50. Reported times are in seconds.
